# Centering and flourishing: an online intervention study assessing the effects of a Christian contemplative practice on stress-reduction and human flourishing

**DOI:** 10.1186/s40359-024-01836-0

**Published:** 2024-07-01

**Authors:** Julia S. Rohde, Sean Goldy, Marianna Graziosi, Michael A. Ferguson, Johannes Thrul, David B. Yaden

**Affiliations:** 1 Department of Psychiatry and Behavioral Sciences, Center for Psychedelic and Consciousness Research, Johns Hopkins School of Medicine, Baltimore, MD USA; 2 Department of Clinical Psychology, Hofstra University, Hempstead, NY USA; 3 Department of Neurology, Harvard Medical School, Boston, MA USA; 4 Center for Brain Circuit Therapeutics, Brigham and Women’s Hospital, MA Boston, USA; 5 Department of Mental Health, Johns Hopkins Bloomberg School of Public Health, Baltimore, MD USA; 6 Sidney Kimmel Comprehensive Cancer Center at Johns Hopkins, Baltimore, MD USA; 7 Centre for Alcohol Policy Research, La Trobe University, Melbourne, Australia

**Keywords:** Centering prayer, Centering practice, Human flourishing, Mindfulness-based interventions (MBIs), Longitudinal online survey design, Mixed-effects modeling

## Abstract

Despite widespread engagement in contemplative religious practices, comparatively little research has been conducted on their potential effects on well-being. Furthermore, few studies have focused on how an explicitly religious framing may impact the outcomes of such practices. In this online randomized controlled trial (*N* = 702), we tested the well-being effects of a contemplative prayer practice called Centering Prayer on self-identifying Christians. We compared 1) presenting the practice with an explicitly religious framing (experimental condition), 2) presenting the practice without an explicitly religious framing (active control), and 3) presenting simple instructions to reflect on the day, without any instructions regarding a meditation-like practice (passive control). After randomization into one of these three conditions, participants were asked to complete their assigned practice daily for 28 days. We hypothesized that the religious framing version of the practice would increase well-being over the active and passive control conditions. Well-being was assessed at three follow-up time points: one day, one week, and one month after the practice period. We found *no* group differences between the conditions on our primary outcome measure of well-being at one-week post-intervention. Each group increased in well-being from baseline to follow-up. We found significant group differences on acute measures of spiritual experience, the Mystical Experience Questionnaire (MEQ-30) and Daily Spiritual Experience Questionnaire (DSES). These results suggest that a religious framing may* not *enhance well-being effects but may alter spiritual outcomes related to contemplative practices.

**Supplementary Information:**

The online version contains supplementary material available at 10.1186/s40359-024-01836-0.

## Introduction

Research on mindfulness, a secularized meditation practice often derived from Buddhist and Hindu roots, has demonstrated some efficacy for enhancing aspects of mental health and well-being (e.g., stress reduction, pain management) and is often recommended in contemporary healthcare settings (for a review, see [27]). Numerous other explicitly religious exercises exist that likely benefit mental health and human flourishing, but rigorous and controlled studies of explicitly religious practices and their effects on general well-being has been relatively limited [77]. This may be a missed opportunity, as it is possible that when concentration practices are combined with the meaning derived from one’s worldview, a synergistic effect will result [9]. Furthermore, a Pew study finds that 55% of the US population already engage in some form of daily prayer practice [59], suggesting that many people are open to engaging in such exercises.

The Centering Prayer is a relatively widespread religious practice that emerged from Christian (especially Catholic) contemplative traditions [32]. The practice was partially inspired by a statement from the Second Vatican Council, which encouraged reviving Christian contemplative practices for use in contemporary society. Trappist priest Thomas Keating is generally acknowledged as the figure who most popularized the Centering Prayer in the 1970s. In describing the historical Christian roots of this practice, Keating cited *The Cloud of Unknowing* as well as writings by St. John of the Cross, St. Teresa of Avila, and Thomas Merton about meditation-like forms of prayer [25, 35]. Centering Prayer is a worldwide Christian practice [49], although prevalence is not precisely known.

The primary intention of Centering Prayer is religious, as it focuses on the practitioner’s sense of connection to God––but it may also increase human flourishing. Flourishing, a category larger than and inclusive of well-being, has been defined as “doing or being well in the following five broad domains of human life: (*i*) happiness and life satisfaction; (*ii*) health, both mental and physical; (*iii*) meaning and purpose; (*iv*) character and virtue; and (*v*) close social relationships” ([65], p. 8149). In the present study, flourishing was operationalized using the Flourishing Measure [65], which has been recommended as a brief and valid outcome measure for assessing flourishing [66].

Specifically, Centering Prayer involves choosing a religious word to silently contemplate, with an intention to invite and become aware of God’s presence [49]. The practice has the virtue of being well-codified and simple to explain. Pennington ([49], p. xvi) describes the instructions of Centering Prayer as follows:

1. *Choose a sacred word as the symbol of your intention to consent to God’s presence and action within.*
2. *Sitting comfortably and with eyes closed, settle briefly and silently introduce the sacred word as the symbol of your intention to consent to God’s presence and action within.*
3. *When you become aware of anything, return ever so gently to God, using the sacred word.*
4. *At the end of the prayer period, remain in silence with eyes closed for a couple of minutes.*

Centering Prayer, like mindfulness, includes a concentration component. But unlike mindfulness, this religious exercise also leverages the sense of meaning derived from one’s religious worldview. Therefore, the main components of this practice involve: 1) a silent meditation with mindfulness-like qualities, and 2) reflection on a religiously meaningful word. We review research relevant to both components below.

### Mindfulness practice

Mindfulness, as conceptualized and constructed in current clinical research trends, is commonly understood to be a form of mental training that promotes attentional control, self-awareness, and emotional self-regulation, which can also sometimes result in self-transcendent experiences [64, 74]. As an intervention, individuals are generally taught to [1] be present in the moment; [2] acknowledge their own thoughts and feelings, however fleeting they may be; [3] accept those thoughts and feelings as momentary and take time to process them with an open mind before reacting [10, 56, 57]. Ultimately, the goal of Mindfulness-Based Interventions (MBIs) is to teach individuals to respond skillfully to mental processes that may contribute to emotional distress and maladaptive behavior, rather than continue to engage in a cycle of behavior that may perpetuate their psychopathology [10].

The efficacy of MBIs has been tested among both clinical and non-clinical populations with generally positive results, albeit of a modest magnitude. The most consistently documented effects of MBIs are reductions in stress, anxiety, and depression symptoms; there have also been observations of increased quality of life and self-compassion [10, 13, 32, 36, 37, 40, 57]. The potential scalability and cost-effectiveness of these interventions make them an important area of research, as access to mental health care remains limited [36]. A better understanding of the mechanisms underlying the positive effects of MBIs may allow for more effective development and delivery of treatment. A systematic review by Gu and colleagues [29] found evidence for cognitive and emotional reactivity and flexibility as underlying mediators of MBI treatment outcomes. Further research is required to evaluate the various modes of MBI and the methods of administering these interventions.

In secular mindfulness practice, the type of concentration and attention required is said to be an ‘open awareness’. Centering Prayer is commonly thought to instead require a form of ‘focused attention’ [23].While the type of attention that secularized mindfulness practice involves may be different from that of contemplative prayer, both the ‘open awareness’-promoting forms of mindfulness and the ‘focused attention’ brought on by Centering Prayer may bring about a similar quieting of the mind [19]. Thus, while the object or form of attention may differ, there are similarities between mindfulness and Christian contemplative practice. Furthermore, pilot studies of Centering Prayer have demonstrated that Centering Prayer may increase mindfulness [15, 23], demonstrating that there may be some similarities in addition to differences.

### Outcomes of religious and secular practices

The primary intention of Centering Prayer in religious contexts is to cultivate a stronger faith relationship. The instructions for this practice were formulated to provide contemporary Christians with a straightforward contemplative daily practice [72]. Centering Prayer, like secular mindfulness practices, may increase well-being. Previous research has shown associations between well-being and various religious practices. A meta-analysis of 19 studies comparing Transcendental Meditation (TM) to non-religious/spiritual forms of meditation showed that TM had greater enhancements in terms of reducing anxiety and drug use as well as increasing positive mental health on measures of self-actualization than non-religious/spiritual practices [1]. A recent meta-analysis of prayer-based interventions suggested that participatory prayer as an adjunct to standard pain treatment may further reduce pain intensity among those with pain conditions or undergoing painful procedures [31]. Christian prayer practices have also been investigated, though to a lesser extent (see [39, 71]).

While the original formulation of the Centering Prayer is in fact religious, there is some evidence to suggest there are significant well-being and health-related effects. Centering Prayer has been investigated in an uncontrolled study among parishioners and was found to reduce levels of stress and anxiety from baseline to post-intervention [19]. Another uncontrolled pilot study among women diagnosed with cancer showed improvements in well-being and reductions in anxiety after engaging with Centering Prayer [31]. These initial studies provide promising data to justify a better powered and more rigorously designed study on Centering Prayer.

Centering Prayer is an explicitly religious practice (i.e., it involves repeating a religious word and inviting God’s presence), *but it is unknown to what extent the religious aspects of the practice contribute to its potential effects on well-being*. Previous research has investigated whether a religious framing of practices contribute to their benefits, with some research showing that an explicitly religious framing of meditation practices results in improved outcomes [67]. Wachholtz and Pargament compared a religiously framed meditative practice compared to a non-religious version of the same practice. The study included an additional control, a general relaxation group. The study found that the group with the religious framing of the meditation reported greater decreases in anxiety, increases in positive mood, closeness to God and significantly more daily experiences of a spiritual nature. This group also displayed increased pain tolerance in a behavioral task. A more recent study by Wachholtz and colleagues [69] investigated the effect of different types of meditation (religious versus non-religious) on migraine headache medication use. The study found that those who practiced Christian-specific meditation had the largest reduction in migraine medication use compared to those who practiced secular meditation or relaxation.

However, not all studies on meditation and/or stress-reduction practices have shown enhancements from a religious framing. Contradicting the findings of Wachholtz and Pargament described above, Feuille and Pargament [21] found that secular meditation significantly reduced pain-related stress relative to simple relaxation, but integrating spirituality into the meditation did not enhance these outcomes. Similarly, an earlier study found that relaxation training and daily prayer result in similar decreases in subjective stress, though only relaxation training outcomes reached the level of statistical significance relative to their control [17]. In a study on the effects of prayer and meditation practices on psychological adjustment [42], the authors found prayer significantly related to self-reported happiness, while secularized meditation and related mindfulness practices (focused attention, open monitoring, and compassion meditation) impact a larger range of positive psychological adjustment outcomes including self-regulation processes of negative emotions.

### The present study

Given the mixed findings in the extant literature, the purpose of this study was to determine whether an explicitly religious contemplative practice would enhance the well-being benefits obtained from a non-religious analog of the same practice and a passive control. Centering Prayer is thought to involve a ‘quieting of the self’ and focused attention on God and/or religious beliefs more generally. Participants are directed to bring their attention to the religious word of choice and notice when their thoughts drift from this term, as a reminder to bring themselves back to the term and the focus on this prayer practice. As such, Centering Prayer combines the benefits of attentional practices with religious meaning and value [9]. This may result in a synergistic effect, which is why we undertook this study to evaluate the potential enhancement offered by integrating religious value into a mindfulness-like practice. We hypothesized that, compared to controls, the explicitly religious version of the practice would result in increased well-being, as measured by the Flourishing Measure [65] over and above those of the active control (secular/neutral word version of the practice) or the passive control.

We had secondary hypotheses that the explicitly religious intervention (experimental) group would outperform the active control group on daily measures of affect and health. Specifically, we hypothesized that the experimental group would demonstrate significantly elevated daily affect and improved health behaviors over the final two weeks of the intervention period when compared to both the active and passive control groups. We further hypothesized the active control group would outperform the passive control group on the same outcomes. The duration of mindfulness interventions varies widely, from a one-time intervention to weeks or months long [26]. As such, there is a limited foundation for an estimate of timeline of effects. However, one early randomized control trial of a two-week contemplative prayer intervention produced significant stress reductions at the end of the two-week intervention [38], providing some precedent for our timepoint assessment.

Our daily measures of health behaviors included exercise, sleep, and socialization. We hypothesized that the explicitly religious intervention would increase movement (i.e., steps), sleep, and socializing compared to the active and passive control conditions. Movement, sleep, and communication have been shown to be important predictors of well-being [16]. Pilot studies of Centering Prayer indicate that this contemplative prayer practice enhances mindfulness [15, 24], which is linked to improved sleep quality [11, 26] and increased physical activity [61]. Furthermore, prior research has revealed a positive relationship between spirituality and religiosity with awe experiences [34]. Awe experiences have been shown to enhance pro-social behavior and social networks [3, 42, 50, 76], constituting an additional route through which the potential additive benefits an explicitly religious mindfulness-like intervention may be exerted. We expected that enhanced well-being post-intervention may be positively correlated with and/or mediated by improved health behaviors over the course of the intervention itself.

Similarly, we predicted that the explicitly religious intervention group would demonstrate increased positive affect on self-report measures over the same time frame (the final two weeks of the intervention period). Tertiarily, we expected individuals in the experimental condition (completing the Centering Practice with an explicitly religious framing) would report a higher incidence of mystical, spiritual, and awe experiences over the course of the intervention period compared to the active and passive control conditions. Early studies suggest that spiritually-integrated meditation practices enhance spiritual health, increase the frequency spiritual experiences, and improve existential well-being relative to secular meditation practices [67, 68]. Relatedly, a study of Centering Prayer found that the prayer practice leads to a deepened and more collaborative relationship with God [24]. We included some other measures of experiences and beliefs for exploratory purposes.

Prior to recruiting participants, our hypotheses and analysis plan were pre-registered via Open Science Framework (OSF): 10.17605/OSF.IO/MC9YA. This study was approved by the Johns Hopkins School of Medicine IRB (IRB00298302).

## Method

### Participants

Participants were recruited using Qualtrics for a study focused on stress reduction to mask our primary outcome (well-being). Because Centering Prayer is explicitly derived from Christian tradition, we recruited only self-identifying Christians for this study. To qualify, participants were required to be 18 years of age or older, affiliate as a Christian, rate higher than neutral (slightly religious or above) on a measure of religiosity and indicate an interest in engaging in a twenty-minute practice daily for four weeks. Participants were also required to own a Smartphone to increase the likelihood that participants would consistently respond to the daily surveys.

#### Participant characteristics

We recruited participants in accordance with our preregistered target enrollment number and estimated drop-out rates. Our study demonstrated higher than expected rates of retention, resulting in a sample size exceeding our initial goal. This yielded a sample size of *N* = 908, with 246 passive control, 344 active controls, and 318 participants in the experimental group. We submitted our inclusion criteria to our subcontracted recruitment platform, Qualtrics, prior to launching our study. Participants were informed of these participation requirements prior to participating. Specifically, participants were informed that they are required to complete a minimum of 18 daily surveys over the course of the 28-day intervention, as well as the one-day and one-week follow-up surveys. Participants who failed to complete one-week follow-up were excluded from analyses because this was the timepoint we specified for our primary outcome measure. These were used as data inclusion criteria in addition to manipulation checks that participants were not directly informed of. These additional manipulation checks required that participants report completing their assigned practice at least five times per week and report an increased frequency of stress-reduction practice (relative to baseline self-report) at one-day follow-up, although we note that some participants who are already doing a maximal amount of stress reduction could have been inadvertently excluded due to this criterion. Participants who did not meet these data inclusion criteria were excluded from further analyses, as they were considered to not have adequately participated in the intervention. For more information on selection for inclusion in data analyses, see Figure A2.

Participants who met inclusion criteria yielded a sample size of *N* = 908, with 246 passive control, 344 active controls, and 318 participants in the experimental group. However, we only report the results of our pre-registered sample size in the main text (results from the full sample are reported in Supplementary Material B and they are highly consistent with those from the pre-registered sample). Adhering to our preregistered plans for analysis, we selected the first 234 participants from each group for analysis. As described, we conducted the same analyses on the pre-registered sample and the full sample, and the results did not differ substantially between the two samples (see Supplementary Material B for results from the full sample). The demographics of the preregistered sample are summarized below (Table 1).

**Table 1:** Demographic Information for the sample (*N* = 702)

Variable	Average Value
Age (SD)	44.92 (24.31)
Extent Religious (SD)^c^	3.09 (0.69)
Sex (% female)	467 (66.52%)
Ethnicity (% Hispanic)^b^	58 (8.26)
Race (count (% of sample))^a^	
Black	140 (19.94%)
White	517 (73.65%)
Multi-racial/Other	45 (6.41%)

^a^Race is grouped into three categories above but, when included in regressions, was necessarily dichotomized (white race versus other) given the skew of our sample. ^b^Ethnicity reflects Hispanic versus non-Hispanic. ^c^Extent religious was measured on a self-reported scale from 0–4 (“Not religious” to “Very religious”), with higher scores indicating a higher extent to which a participant identified as religious and a value of three most closely aligning with the response, “Moderately religious” on this scale. No significant between-group differences were found among these demographic variables at baseline. *SD* Standard Deviation. Individuals were randomized to an assigned behavioral intervention, and equal-sized behavioral intervention groups were extracted after data processing (*N* = 234) based on earliest completion of survey study

### Materials & measures

#### Pre-cursor centering words survey: determining “Neutral” and “Sacred” Words

To provide sacred terms (for the experimental condition) and neutral/secular terms (for the active control condition) for the current study, we previously surveyed Christian-identifying adults to determine a list of ten “sacred” words and a list of ten “neutral” words around which the experimental and active controls would use in their practice. Results from this sub-study are available in the supplementary materials (see Table A8).

#### Baseline and follow-up surveys

Participation in the study involved a 60-day commitment, beginning with a baseline survey, followed by 28-days of daily practice (behavioral intervention) and brief surveys, then follow-up surveys at day 29, 35, and 60 (one-day, one-week, and one-month after the 28-day intervention period). Table 2 reports the timing and frequency of each of these surveys, as well as the scales included in each. These scales are described in greater detail below.

**Table 2:** Schedule of assessments administered via online surveys

Baseline	Daily During 28-Day Intervention Period	One-day Post-Intervention	One-week Post-Intervention	One-month Post-Intervention
Flourishing		Flourishing	*Flourishing*	Flourishing
ESAT (full)		ESAT (full)	ESAT (full)	ESAT (full)
AWE-S		AWE-S		
	Happy (ESAT item)	DSES	DSES	DSES
	Sad (ESAT item)	MEQ-30		
	Sleep; Physical Activity; Social interaction (rotating)	PI-6	PI-6	PI-6
		Philosophical Beliefs	Philosophical Beliefs	Philosophical Beliefs
		Belief Changes	Belief Changes	Belief Changes
	Journal entry (*optional*)			

Scales and/or items administered at Baseline (pre-intervention); throughout the 28-day intervention period; and post-intervention (one-day, one-week, and one-month post-intervention). Items and assessments included above are as follows: Flourishing Scale (“Flourishing”; italics reflect primary outcome time point), Emotional State Assessment Tool (ESAT), Awe Experience Scale-Short Form (AWE-S); [Intervention Period] “Happy” and “Sad” refer to single-item questions extracted from the ESAT to reflect daily self-assessments of affect and emotional state; “Sleep”, “Exercise”, and “Social Interaction” are single-item questions administered on a rotating basis (each item administered once every three days) to assess health behaviors, Mystical Experience Questionnaire (MEQ-30), Daily Spiritual Experience Scale (DSES), Primals Inventory (PI-6), Philosophical Beliefs Scale, and Belief Changes Scale

#### Flourishing measure

The flourishing measure [65] is a 12-item measure of well-being across six domains: 1) Happiness and Life Satisfaction, 2) Mental and Physical Health, 3) Meaning and Purpose, 4) Character and Virtue, and 5) Close Social Relationships. The optional sixth Financial and Material domain was not used in this study. This measure demonstrated reliable internal consistency (α = 0.93).

#### Emotional state assessment tool (ESAT)

The full 18-item ESAT scale [75] was administered at baseline and all three follow-up timepoints. The ESAT is a measure of affect, with a classic two-dimensional structure: 1) positive emotion and 2) negative emotion. This measure demonstrated internal consistency (ESAT Positive Emotion, α = 0.92; ESAT Negative Emotion, α = 0.93).

#### Awe experience scale (AWE-S)

This scale measures the overall intensity and individual aspects of the experience of awe [76]. Awe has been associated with improved well-being. Correlational work has associated more incidences of awe with increased well-being [2, 4, 54]. Experimental work inducing awe has also observed increases in well-being outcomes [3, 33, 50]. The AWE-S includes 6 factors: 1) perception of vastness, 2) need for accommodation, 3) altered sense of time, 4) altered sense of self, 5) connectedness, and 6) physiological changes. The AWE-S Short Form (6-items) was used in the present study, consisting of the top loading items in each of the six factors in the original AWE-S scale. This measure demonstrated internal consistency (α = 0.66).

#### Daily spiritual experience scale (DSES)

This 16-item self-report measure [63] assesses connection with the transcendent in daily life—the ordinary experiences of spirituality such as awe, joy that lifts one out of the mundane, and a sense of deep inner peace. This measure demonstrated internal consistency (α = 0.96).

#### Mystical experience questionnaire (MEQ-30)

This 30-item scale [8] assesses multiple components of altered states of consciousness commonly associated with psychedelic experiences. The MEQ-30 is comprised of four factors: unity, positive mood, space/time, and ineffability. This measure demonstrated internal consistency (α = 0.96).

#### Primals inventory (PI-6)

This six-item scale [14] asks about basic beliefs about the world (e.g., “Most things in the world are good”). This measure demonstrated internal consistency (α = 0.81).

#### Philosophical beliefs survey

This scale explores questions related to one’s philosophical beliefs as part of a larger effort to better understand the effects of those beliefs on our lives. Four Philosophical Beliefs items [73] were included in the present study, assessing beliefs regarding the following philosophical domains: Aesthetic Value (Beauty), Free Will, God, and Mind. Participants were presented with 2–3 pre-configured, opinions/statements about the philosophical domains listed above and asked to indicate which opinion/statement aligned most closely with their personal belief. Each item was considered and analyzed separately, and internal consistency was not assessed.

#### Belief changes

The six items included in this exploratory measure [45] investigate the participant’s basic beliefs about consciousness and meta-physics (e.g., “The mind is not part of the brain, but it affects the brain”). The first item was derived from the Reflective Dualism subscale of the Mind–Body Relationship Scale [53]. The wording of items 2, 3, 4, and 6 were used previously by Nayak and colleagues [45]. The fifth item was derived from the Revised Paranormal Belief Scale [62]. The six items included as part of this exploratory measure were considered and analyzed individually. Internal consistency was not assessed.

#### Daily surveys

Three questions, in additional to an optional journal entry, were administered daily during the practice period. The first two questions were extracted from the Emotional State Assessment Tool (ESAT; 76) and administered daily. The third question was a self-report of health behaviors, with a single item about sleep, exercise, and social activity administered on a rotating basis (every three days).

1. I feel happy. (“Does not apply at all” to “Completely applies”)
2. I feel sad. (“Does not apply at all” to “Completely applies”)
3. Rotation of the following three items (once every three days):
  i. How much did you sleep last night? (“ < 4 h” to “8 + hours”)
  ii. How much low-intensity exercise (walking, jogging, moving around) did you engage yesterday? (“None” to “3 + hours”)
  iii. How much time did you spend engaging socially (in-person, over the phone, etc.) yesterday? (“None” to “4 + hours”)

In the present study, daily affect balance was calculated by averaging the first two questions, positive affect item together with the negative affect item (reverse scored) and analyzed by plotting time-course data and fitting appropriate models to investigate how these variables change over the course of the intervention period across groups. Per our preregistration, we provide a supplemental analysis of daily affect balance dichotomized across the intervention period by averaging it over the first (Days 1–14) and last (Days 15–28) two weeks of the intervention period, as we hypothesized that the effects of the behavioral intervention will be evident after approximately two weeks.

#### Written journal entries

Written journal entries were also collected from participants via daily and follow-up surveys. These entries were optional, offering participants an open-ended way to describe and reflect on their daily life and experiences, the content of which is not the focus of the present research.

#### Smartphone remote sensors

Survey findings were complemented by direct monitoring of behavioral outcomes (accelerometer, distance travelled, steps, elevation/stairs climbed, number of minutes spent using call function, device usage) via the AWARE application [20]. Per our preregistration, these data were collected to complement the self-report measures of behavioral health activities and provide an additional measure of well-being, as movement, sleep, and communication have been shown to be important predictors of well-being [15]. Due to low adherence to use of the mobile application amongst other technological issues, these data are not included in this report.

Participants were told during recruitment that they would be asked to engage in a stress-reduction practice for 20 min a day for four weeks to examine the effects of this practice on stress. Participants were not told the exact details of the different versions of the practice or that the purpose of the study was to assess increases in well-being. All participants received daily reminders to engage in their assigned practice and respond to daily survey links (via email and/or text) throughout the 28-day practice period, as well as reminders to complete the follow-up surveys at one day, one week, and one month after the practice period. Participants who enrolled in the study were eligible to earn up to $55 for their participation in the study.

Participants deemed eligible were sent an invitation to enroll in the study. This study was approved by the Johns Hopkins Medicine Institutional Review Board. Participants were randomized into one of three conditions—the experimental condition were instructed to engage in Centering Prayer, the active control condition engaged in what we call Centering Practice (i.e., a modified version of Centering Prayer, with all religious terminology replaced by neutral/secular language), and the passive control was asked to simply reflect on their day and respond to the daily surveys (see Table 3 for instructions provided for each condition). Two control conditions were included with the intent to detect whether Centering is effective at all (inferred from comparing the experimental and active control groups to the passive control group) and whether Centering is enhanced by religious/spiritual meaning (inferred by comparing the experimental group to the active control group). Limitations of the study design are described in the discussion section.

**Table 3:** Behavioral intervention instructions provided by group

Experimental Condition	Active Control	Passive Control
Each day, for about 20 min a day, your practice is to do the following: 1. Choose a **sacred word from the list below** as the symbol of your intention to consent to God’s presence and action within 2. Sitting comfortably and with eyes closed, settle briefly and silently introduce the sacred word as the symbol of your intention to consent to God’s presence and action within 3. When you become aware of anything, return ever so gently to God, using the sacred word 4. At the end of the prayer period, remain in silence with eyes closed for a couple of minutes	Each day, for about 20 min a day, your practice is to do the following: 1. Choose a **word from the list below** as the symbol of your intention 2. Sitting comfortably and with eyes closed, settle briefly and silently introduce the word as the symbol of your intention 3. When you become aware of anything, return ever so gently to your practice using the word 4. At the end of the practice period, remain in silence with eyes closed for a couple of minutes	Each day, for about 20 min a day, your practice is to do the following: 1. Take note of your daily life and what happens to you 2. Take daily survey (via link sent by email and/or text)
Please choose a word from the following list as your symbol of intention: • God • Jesus Christ • Sacred • Heaven • Bible • Holy • Angel • Amen • Bless • Soul	Please choose a word from the following list as your symbol of intention: • Ball • Ticket • Furniture • Yard • Pillow • Bed • Eleven • Coffee • Bike • Soap	
Please remember to **complete your daily check-ins**. **These will take approximately 1-min to complete**	Please remember to **complete your daily check-ins**. **These will take approximately 1-min to complete**	Please remember to **complete your daily surveys. These will take approximately 1-min to complete**

Participants were assigned to one of three conditions using simple randomization via Qualtrics. The behavioral intervention consisted of three conditions, 1) experimental (centering prayer, using a religious/spiritual word), 2) active control (centering practice, using a neutral word and no religious content), and 3) passive control c (daily measures and no practice other than instructions to pay attention to daily life). The experimental and active control groups were asked to choose a sacred and neutral word, respectively, the choices for which were determined by a precursor survey described previously

### Analytic approach

Pairwise comparisons of multivariate regression models across groups were conducted with Tukey's family-wise adjustment to control for multiple comparisons. Covariates for multivariate regressions consisted of all available demographic and related variables collected at baseline. Group (behavioral intervention), sex, race (dichotomized as a categorical variable to reflect white race or other, given the skew of our sample), and ethnicity (Hispanic or non-Hispanic) were treated as categorical variables in regressions. Self-reported religiosity (slightly religious, moderately religious, or very religious) and age were treated as continuous variables. When available, baseline scores were always included as covariates in the multi-variate regression models. When available, baseline scores were always included as covariates in the multi-variate regression models. The inclusion of the above covariates as part of our approach was based on the precedent set by VanderWeele—the author of the Flourishing Measure, operationalized as our assessment of well-being— and his colleagues [11]. No between-group differences in any of the variables included as covariates were found at baseline. This approach was used to conduct comparisons across groups for all post-intervention (follow-up) outcomes.

In modeling daily survey outcomes over the course of the intervention period, we generated a linear mixed-effects model fitfor each daily outcome variable (self-reported daily affect balance, sleep, exercise, and social engagement). To account for repeated daily observations and for random differences in participants' outcome trajectories over time, each model includes a random intercept and slope, respectively, as well as a group by time interaction. The linear mixed-effects model allows us to assess changes in outcomes over time by group while accounting for the nested, repeated observations within participants. In assessing within-group changes over time, we stratified by group and examined the simple slopes of time for each outcome. Per our preregistration, we also compare the daily survey outcomes by averaging the self-reported values over the dichotomized intervention period (days 1–14 and 15–28), hypothesizing that it may take about two weeks for the intervention to impact affect balance and health behaviors. We have included these results in the supplementary materials (see Figure A1, Table A5).

In analyzing and reporting outcomes, the threshold of statistical significance was *p* < 0.05 and CI refers to 95% confidence intervals. Exploratory analyses are represented as such.

## Results

### Primary outcome: flourishing

Our primary hypothesis was *not *supported, as we did not observe significant between-group differences in flourishing scores at one-week after the practice period (one-week post-intervention). No significant differences were found between groups at the primary outcome timepoint (one-week post-intervention): Experimental versus Active (*b* = 0.04, *SE* = 0.12, *t*(693) = 0.32, *p* = 0.95, *d* = 0.03); Experimental versus Passive (*b* = 0.23, *SE* = 0.12, *t*(693) = 1.97, *p* = 0.12, *d* = 0.18); Active versus Passive (*b* = 0.19, SE = 0.12, *t*(693) = 1.64, *p* = 0.23, *d* = 0.15). Between-group differences in our primary outcome measure, total flourishing, were neither found at any other post-intervention timepoints (Supplementary Table A2) and no between-group differences were found at baseline. These results were replicated in the full sample. Full sample results can be found in tables and figures in the supplementary materials (Supplementary Material B).

**Fig. 1:**
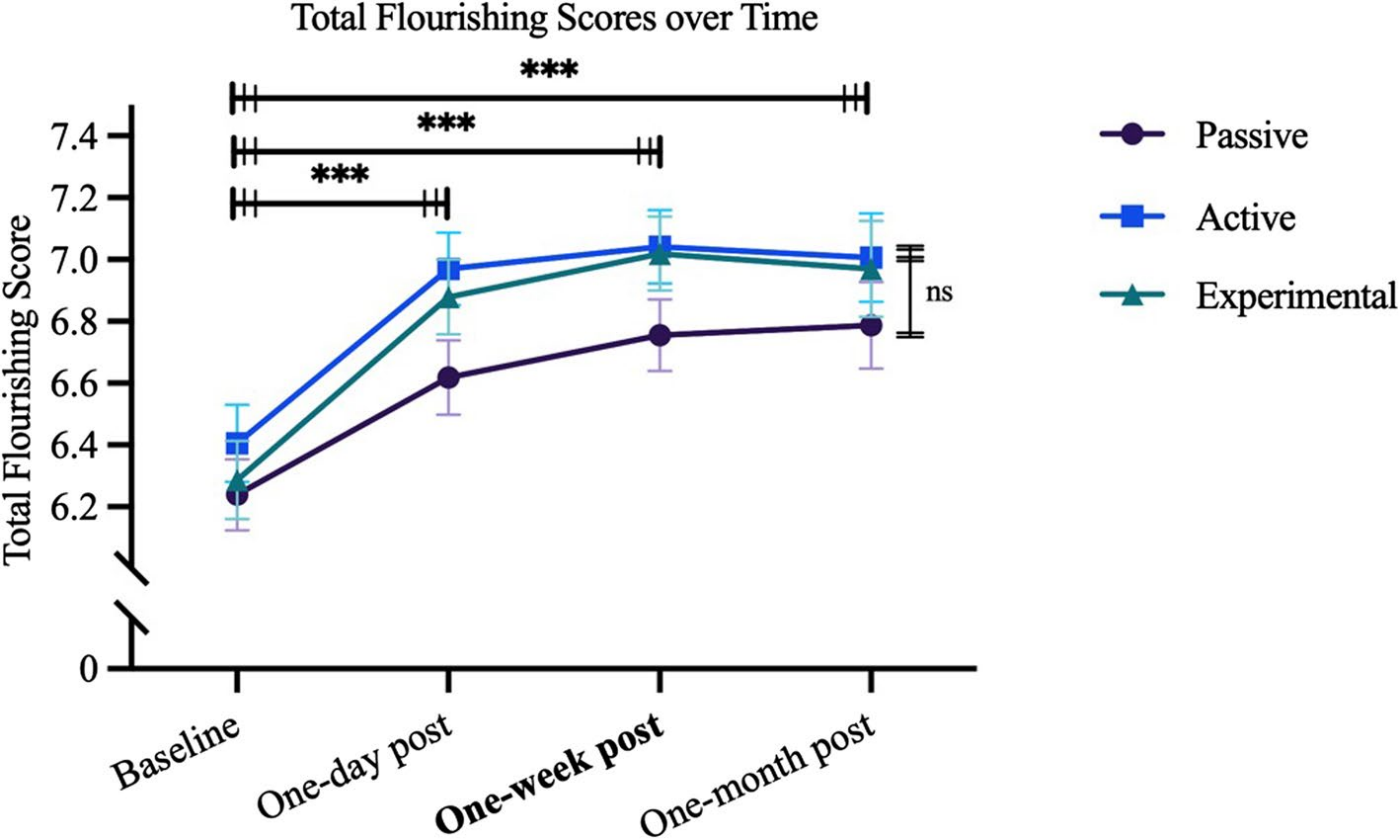
Total flourishing scores plotted over time, by group. Note. Results of pairwise comparison (on right side of the graph) and paired t-tests (displayed horizontally, on top of the graph) are displayed (ns = not significant; ***p < 0.001). For pairwise comparisons, a Tukey family-wise adjustment was applied. No significant between-group differences were found in our primary outcome, total flourishing. Covariates included in the regressions compared pairwise included group, sex, age, race, ethnicity, self-reported religiosity, as well as baseline total flourishing scores (no between-group differences found at baseline). Horizontal bars with triple dashes at each end signify that the asterisk placed above the bar reflect results of paired t-test analyses conducted within all three groups from baseline to each follow-up timepoint. Range of total flourishing score values is 0–8. Error bars represent standard error

As an additional analysis of our primary outcome measure, we conducted paired t-tests to determine the changes in flourishing scores over time. We assessed these changes from baseline to one-week post-intervention within each group (see Fig. 1). We found significant increases in total flourishing scores from baseline to one-week post-intervention within each group. Increases among the Passive control group from baseline (M = 6.24, SE = 0.12) to one-week post-intervention (M = 6.76, SE = 0.12) were significant, CI = [0.33, 0.7], *t*(233) = 5.52, *p* < 0.001, *d* = 0.29; similar significant increases were observed in the Active control group from baseline (M = 6.41, SE = 0.13) to one-week post (M = 7.04, SE = 0.12), CI = [0.45, 0.82], *t*(233) = 6.86, *p* < 0.001, *d* = 0.34; and in the Experimental group from baseline (M = 6.29, SE = 0.13) to one-week post (M = 7.02, SE = 0.12), CI = [0.57, 0.9], *t*(233) = 8.76, *p* < 0.001, *d* = 0.39 (see Table A3). Total Flourishing scores for each group remained relatively stable across the one-day, one-week, and one-month post-intervention follow-ups. Summary values of total flourishing scores at all post-intervention timepoints are summarized in (see Table A1). The significant differences found by comparing baseline and one-week post-intervention via paired t-tests were similar at the one-day and one-month post-intervention time points.

### Secondary outcomes: affect balance and health behaviors

**Fig. 2:**
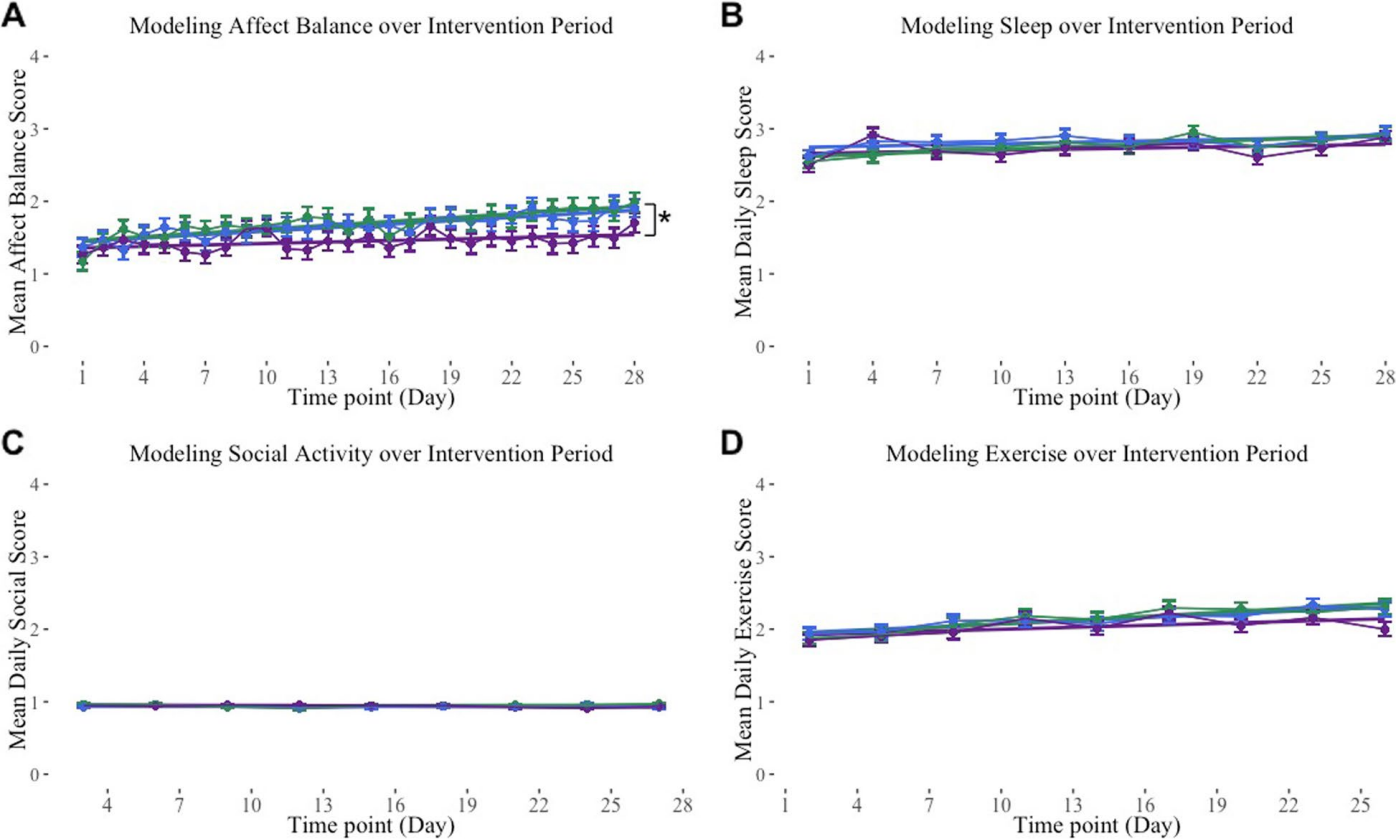
Linear mixed-effects model of self-reported daily affect balance, sleep, exercise, and social engagement over the intervention period, by group. Note: Error bars represent standard error. **A** Comparisons of the affect balance models revealed significant differences between only the Experimental and Passive groups over time (*p < 0.05). For pairwise comparisons, a Tukey family-wise adjustment was applied. Daily affect balance scores range from -4 to 4. **B**, **C**, **D** Linear mixed-effects model of self-reported sleep, exercise, and social engagement over the intervention period, by group. No significant between-groups were found on these behavioral outcomes. Mean daily sleep, exercise, and social engagement scores range from 0 to 4

We investigated the between-group differences in self-reported affect balance, defined by two items extracted from the ESAT (composite score of response values to happiness and sadness). We estimated linear mixed-effects models of self-reported affect balance over the course of the intervention period by group, noting significant differences in the group by time interaction term. We plotted the mean affect balance by group and day, overlayed with the linear model that best fit the data for each group, to demonstrate how this outcome changed over time by group in each sample (see Fig. 2A). Our analyses revealed significant differences between the Experimental and Passive groups over time (*b* = 0.01, *SE* = 0.01, *t*(18,214) = 2.16, *p* = 0.03). Comparisons of growth models between Active and Passive groups were not significant, nor were comparisons between the Experimental and Active groups.

We examined between-group differences in health behaviors by separately comparing self-reported sleep, exercise, and social engagement, collected via the daily self-report surveys, using the same approach described above. Using the same analyses as for the outcome affect balance, we estimated linear mixed-effects models of these self-reported health behaviors over the course of the intervention period by group, noting significant differences in the group by time interaction term. We plotted the mean self-reported health behavior scores (defined by response values to single-item questions regarding how much sleep, exercise, and social engagement the participant engaged in over the last twenty-four hours, with each single-item health behavior question administered via daily surveys once every three days) by group and day, overlayed with the linear model that best fit the data for each group, to demonstrate how this outcome changed over time by group in each sample (see Fig. 2B, C, D*)*. Our analyses revealed no significant differences in health behaviors between groups.

In addition to analyzing group differences in affect balance and health behaviors, we also examined the fixed effect of time (simple slope, *b*) of group-specific linear mixed-effects of each outcome. These values are compared to a zero slope to generate a *p*-value, indicating whether there was a significant effect of participating in the intervention on the self-reported affect balance and health behavior outcomes (see Table A4). Within the Experimental group, significant effects of time were found on Affect (*b* = 0.02, *SE* = 0.004, *t*(18,214) = 4.92, *p* < 0.001), Sleep (*b* = 0.01, *SE* = 0.003, *t*(6043) = 3.71,* p* < 0.001), and Exercise (*b* = 0.02, SE = 0.003, *t*(5390) = 5.16, *p* < 0.001) outcome models. Within the Active group, significant effects of time were found on Affect (*b* = 0.02, *SE* = 0.004, *t*(18,214) = 4.57, *p* < 0.001), Exercise (*b* = 0.02, *SE* = 0.003, *t*(5390) = 4.84, *p* < 0.001), and (to a smaller but still significant degree) Social (*b* = 0.01, *SE* = 0.004, *t*(5371) = 2.41,* p* = 0.02) outcomes. Within the Passive group, significant effects of time were observed only on the Exercise outcome (*b* = 0.09, *SE* = 0.003, *t*(5390) = 2.7, *p* = 0.007). However, care should be taken in interpreting these results, as the effect of the interaction of time on condition is diminishingly small, especially given the degrees of freedom in this model. Per our preregistration, in addition to analyzing the averages of these self-reported behaviors over the course of the entire intervention period, we averaged these self-report outcomes over the first and the last two weeks of the intervention period (see Figure A1). Our analyses of these dichotomized daily survey outcomes revealed no significant differences between groups; however, significant within-group changes over time on a number of these outcomes were found (see Table A5), largely confirming our mixed-effect model findings.

### Exploratory outcomes

We investigated various measures of experiences that may have occurred during the practice period, which were assessed at the conclusion of the practice period (one-day post-intervention) across groups. We also conducted multivariate regressions and pairwise comparisons to determine whether scores on the ESAT, DSES, and AWE-S at one-day post-intervention differed significantly across conditions and/or across time (compared to baseline, within-group changes). Additionally, we used multivariate regressions and pairwise comparisons to determine if there were significant differences on MEQ scores across conditions at one-day post-intervention as well as where the differences are found across groups. Baseline scores were available for the ESAT and AWE-S measures, and thus were included in the regression models. Demographic factors (age, sex, race, age, and self-reported religiosity) were included as covariates in regressions. For these pairwise comparisons, a family-wise adjustment was applied. Given that no significant between-group differences in our primary outcome were found, we did not conduct mediation analyses of these measures on our primary outcome. All analyses and results below are exploratory and should be understood as such.

**Fig. 3:**
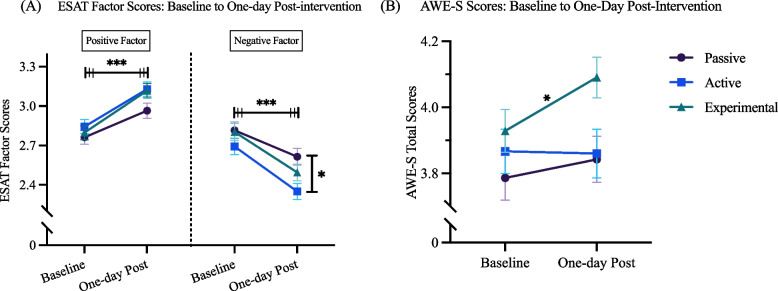
ESAT factor (positive and negative) and AWE-S total scores at baseline and one-day post-intervention. Note: Significant paired t-test results (on the top of the plots) and pairwise comparisons (on the right side of the plots) are marked by an asterisk (*p < 0.05, **p < 0.01, ***p < 0.001). For pairwise comparisons, a Tukey family-wise adjustment was applied. The triple bars over the ESAT factor score plots signify that the asterisk reflects results of paired t-test analyses conducted within each of the three groups. Error bars represent standard error

Pairwise comparisons of regression models and paired t-tests were conducted on exploratory measures that were administered at both baseline and one-day post-intervention, the AWE-S and ESAT outcomes, assessing experiences of awe and changes in emotional states experienced before and after completing the assigned behavioral intervention. Pairwise comparisons of AWE-S total score regressions revealed no significant between-group differences at one-day follow-up (see Table A6). Pairwise comparisons of ESAT factor multivariate regressions revealed a significant difference between the Active and Passive group ESAT negative factor scores at one-day post-intervention (*b* = -0.18, *SE* = 0.06,* t*(693) = -2.87, *p* = 0.01, *d* = -0.27; see Fig. 3, Table A6).

Within-group comparisons of exploratory outcomes between baseline and post-intervention (comparisons on outcomes for which baseline scores were available, ESAT and AWE-S only) were conducted. Paired t-tests revealed significant increases in AWE-S total scores from baseline to one-day post-intervention in the Experimental group only (MD = 0.16, CI = [0.04, 0.29], t(233) = 2.58, p = 0.01; see Fig. 3, Table A7). Paired t-tests revealed significant increases in ESAT Positive and significant decreases in ESAT Negative factor scores from pre- to post-intervention for all groups (see Fig. 3, Table A7). Significant increases in ESAT Positive factor scores over time reflect increased levels of positive emotion and were similarly found across all groups: Passive from baseline (M = 2.74, SE = 0.05) to one-day post-intervention (M = 2.96, SE = 0.06), *CI* = [0.12, 0.29], *t*(233) = 4.66, *p* < 0.001, *d* = 0.24; Active from baseline (M = 2.86, SE = 0.04) to one-day post-intervention (M = 3.15, SE = 0.05), *CI* = [0.2, 0.37], *t*(233) = 6.63, *p* < 0.001, *d* = 0.35; Experimental from baseline (M = 2.79, SE = 0.05) to one-day post-intervention (M = 3.10, SE = 0.05), *CI* = [0.23, 0.4], *t*(233) = 7.41, *p* < 0.001, *d* = 0.38. Decreases in ESAT Negative factor scores reflect decreased levels of negative emotion and were found across groups: Passive from baseline (M = 2.83, SE = 0.06) to one-day post-intervention (M = 2.61, SE = 0.06), *CI* = [-0.3, -0.1], *t*(233) = -4.02, *p* < 0.001, *d* = -0.21; Active from baseline (M = 2.69, SE = 0.05) to one-day post-intervention (M = 2.35, SE = 0.05), *CI* = [-0.43, -0.25], *t*(233) = -7.47, *p* < 0.001, *d* = -0.36; Experimental from baseline (M = 2.82, SE = 0.06) to one-day post-intervention (M = 2.49, SE = 0.06), *CI* = [-0.41, -0.21], *t*(233) = -6.09, *p* < 0.001, *d* = -0.31. These significant increases in ESAT Positive and Negative factors relative to baseline were maintained at one-week and one-month post-intervention across groups (see Table A7).

Paired t-tests revealed significant increases in AWE-S total scores from baseline to one-day post-intervention in the Experimental group only (*MD* = 0.16, *CI* = [0.04, 0.29], *t*(233) = 2.58, *p* = 0.01; see Fig. 3, Table A7).

**Fig. 4:**
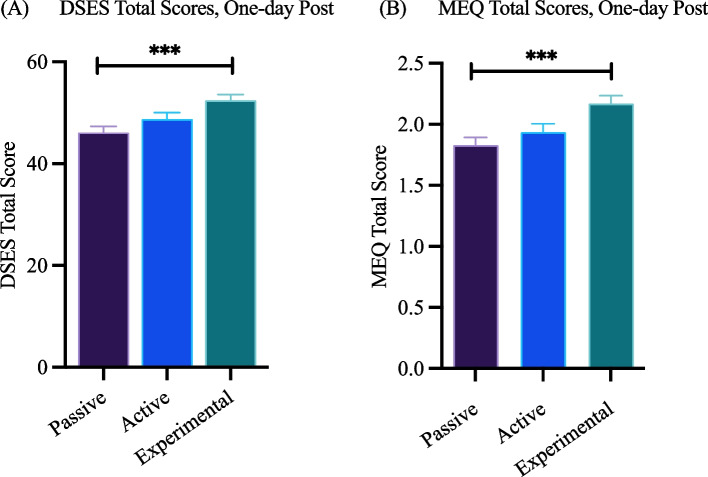
Mean (**A**) DSES and (**B**) MEQ-30 outcomes at one-day post-intervention. *Note:* Significant pairwise comparison results of multivariate regressions are represented by asterisks, ***p < 0.001, and mean values by group are plotted. Regressions included group, sex, age, race (dichotomized), ethnicity, and self-reported religiosity as covariates. For pairwise comparisons, a Tukey family-wise adjustment was applied. Error bars represent standard error

The DSES and MEQ scales were only administered post-intervention and results of between-group comparisons (pairwise comparisons of multivariate regression models) are shown in Fig. 4. Pairwise comparisons of the DSES regression models showed significant differences on DSES total scores one-day post-intervention between the Experimental and Passive groups (*b* = 6.46, *SE* = 1.55, *t*(694) = 4.16, *p* < 0.001, *d* = 0.39; see Fig. 4A, Table A6). Pairwise comparisons of DSES Total score regressions continued to reveal significant differences between Experimental and Passive groups at one-week post-intervention (*b* = 5.04, *SE* = 1.55, *t*(694) = 3.26, *p* < 0.01, *d* = 0.3) and one-month post-intervention (*b* = 5.95, *SE* = 1.82, *t*(478) = 3.27, *p* < 0.01, *d* = 0.36).

Pairwise comparisons of the MEQ regression models revealed significant differences in MEQ Total scores (Fig. 4B, Table A6) between only the Experimental and Passive groups (*b* = 0.35, *SE* = 0.09, *t*(694) = 3.95, *p* < 0.001, *d* = 0.37).

### Additional exploratory outcomes: PI-6, philosophical beliefs scale, and change in beliefs scale

We used the analytic approach described previously to conduct exploratory analyses of the PI-6, Philosophical Beliefs Scale, and Change in Beliefs Scale across groups one-day post-intervention. Analyses were conducted on individual items of the Change in Beliefs and Philosophical Beliefs Scale, while the PI-6 total score was analyzed. Pairwise comparisons revealed no significant differences between groups on these extra-exploratory outcomes.

## Discussion

The present study found that Centering Prayer, a Christian contemplative practice in some ways similar to mindfulness, did *not* enhance flourishing and other health behaviors compared to a non-religiously framed version of the practice and a passive control. We hypothesized that a religiously framed meditation practice would enhance the effects of meditation on well-being, but our hypothesis was not supported, as we found no differences on well-being between a religiously framed meditation practice (experimental condition), a non-religious framed meditation practice (active control), or a condition that asked participants to notice their daily life (passive control). Our secondary hypotheses regarding affect balance and health behaviors (sleep, exercise, social engagement) over the course of the intervention period compared to controls were largely unsupported, apart from one finding of significantly elevated affect balance among the experimental group relative to the passive control over the course of the intervention period. Our results suggest that the integration of religion/spirituality into meditation practices undertaken for the purpose of stress reduction do *not* result in any unique additional benefit to well-being.

Prior research regarding the integration of explicitly religious terms and/or framework into meditation and/or stress-reduction practices is limited and, even where it does exist, has been mixed. Situating our own study in the context of such literature, we note that our results do not align with the primary findings of Wachholtz and Pargament [67, 68], who reported significant improvements in positive emotion and pain tolerance from a spiritual intervention compared to secular and passive control groups.

However, ours is not the first study to demonstrate these findings may not be easily replicated. As described previously, in a study on the effects of a two-week explicitly spiritual mindfulness intervention versus secularized mindfulness and relaxation-only interventions on pain-related outcomes among migraineurs [21], those in the secularized mindfulness group experienced the greatest reductions in pain-related outcomes. Integrating spirituality into the mindfulness intervention did not enhance pain-reduction. Our findings similarly fail to support the idea that integrating religion into mindfulness interventions enhance the well-being effects of those interventions. Similarly, a prior study comparing religious versus secular interventions to promote forgiveness in romantic relationships found that both secular and religious interventions produced similar benefits in forgiveness and well-being relative to a passive control [55]. Integrating religion into the forgiveness-oriented intervention did not enhance the effects. Our findings similarly suggest that an explicitly religious intervention may produce benefits that are specific to religious or spiritual facets of well-being but do not enhance overall well-being and health. We found significant group differences between the experimental and passive conditions in our exploratory analyses comparing mystical and spiritual experiences (MEQ, DSES). On our measure of mystical experiences, the experimental group scored significantly higher than the passive control groups. On our measure of daily spiritual experiences, the experimental group scored significantly higher than the passive control and these significant differences remained at one-week and one-month post-intervention. Similarly, on our measure of awe experiences, only the experimental group demonstrated significant increases on this measure from pre- to post-intervention. These exploratory aspects of our findings align more closely with Wachholtz and Pargament [67] than our primary outcome findings. Wachholtz and Pargament found that their spiritual intervention group increased significantly on measures of spiritual and mystical experience. This may point to the need to include measures more targeted to spiritual and religious facets of well-being in subsequent investigations.

Despite our hypothesis that the experimental group would derive significantly greater spiritual benefits from their explicitly religious intervention than both the active and passive control groups, we found significant differences in spiritual and mystical outcomes only between the experimental and passive control groups. This indicates that the active control group may have derived some spiritual or religious benefit from their secularized intervention. Thus, our findings may provide minor support for previous assertions that meditation practices cannot be ‘secularized’—that is, extracted completely from their religious origins because religious/spiritual participants may inevitably apply this meaning to even secularized practices [12]. In our study, self-identified Christians may be familiar with the Centering Prayer and thus inherently imbue the practice with at least some religious meaning.

Several factors in our study may limit our ability to generalize our findings and/or broadly claim that our null results provide evidence that all explicitly religious or spiritual practices offer no additional benefit to well-being. One reason for the discordant findings in the our study relative to pilot studies of explicitly religious and spiritual interventions by Wachholtz and Pargament [67, 68]— as similarly noted by Feuille and Pargament [21]— may be the phrase on which participants were instructed to focus during their daily practice. Whereas Wachholtz and Pargament instructed their participants to focus on explicitly positive valence attributes of religious belief (i.e., “God is love”), our participants were simply instructed to focus on singular terms related to Christianity (i.e., “Jesus Christ”, “Holy”). It may be worth noting that our study did not assess the self-perceptions of prayer efficacy and importance, which have previously been highlighted as important mediators in the relationship between religious practice and well-being-related outcomes [17]. Moreover, religion and spirituality, though often important in promoting well-being, are not ubiquitously health-promoting factors. The relationship between these factors and well-being are complex and often idiosyncratic (i.e., dependent on personal experience, religious coping styles, feelings of connectedness with the divine, and/or feelings of shame and guilt evoked by religious practice [43, 71]). For example, feelings of connectedness both to a divine being and/or a religious community have been found to contribute to life satisfaction [51], but individuals who may believe in a higher power without feeling connected to that higher power and/or a religious community may be at greater risk for mental health issues [7].

However, unlike the previous studies described above, we did not find significant differences between our Experimental and Active Control groups relative to our Passive Control on our primary outcome measure of well-being. One reason that all three groups demonstrated significant increases in flourishing from pre- to post-intervention may have been due to the inadvertent strength of the intervention assigned to our Passive Control group via daily diary and reflection. While the experimental and active conditions were given explicit instructions regarding what to reflect on during their 20-min practice period (centering themselves on either a religious/sacred or neutral/secular term, depending on condition), the passive group was simply instructed to reflect on their daily life, respond to the daily survey items, and (optionally) journal.

Journaling has been shown to be an effective intervention to enhance well-being (e.g., [28, 41, 47, 48, 60]). Though we cannot liken this broad reflective journaling to a single interventional paradigm, writing about both negative and positive emotions and experiences—expressive writing, narrative writing, or Positive Affect Journaling—have been shown to produce improvements in well-being and reductions in mental distress [28, 41, 58, 60]. This is further supported by the findings of an online RCT conducted by Baikie and colleagues [5], which demonstrated that expressive, positive, and even time management control intervention groups produced improvements on measures of mental and physical well-being. For those in the passive group, who cumulatively wrote almost double that of the experimental and active groups, this may be particularly true and the amount they wrote may have allowed them to better confront their daily stressors and subsequently integrate those events into a cohesive narrative for themselves. Ultimately, this may have made for an effective well-being intervention in what we intended to be a passive control condition. Furthermore, the attention concentration portion of the practice alone (both for active and passive control groups, who were both directed to spend twenty minutes on their practice) may produce well-being benefits. Future studies should consider a truly passive control, who are simply requested to respond to daily survey questions without any suggestion that they conduct daily reflection or given an opportunity to journal. This may help identify what is driving the effects—that is, the lack of significant between-group differences in well-being—that we observe in this study.

### Limitations and directions for future research

The present study was limited in several ways. First, the study was administered online, and while adherence was measured by completion of daily surveys, online intervention and survey studies are inherently vulnerable to high rates of non-compliance [22, 30, 52]. Accountability is difficult to create and adherence impossible to enforce beyond the incentives offered. We sought to identify non-compliant participants using the filters applied for data exclusion, as described earlier. However, it is possible that participants who completed the minimum number of daily and follow-up surveys did not complete the twenty minutes of their assigned behavioral intervention/mediation practice. As noted by Hanano and colleagues [30], numerous reviews of self-guided online intervention studies have reported poor adherence rates, with one meta-analysis reporting an average rate of 26% adherence to a self-guided online intervention for depression [52] and another similar study of online treatment options reporting adherence rates as low as 3.9% [22]. We attempted to address this issue by sending daily reminders. Ultimately, it was not possible for us to assess the degree to which participants completed their assigned behavioral intervention throughout the 28-day practice period. In the future, the addition of some form of interactive guidance, the use of online timers that force participants to measure their own duration of practice, and other methods of increasing objective confirmation of compliance are needed.

Second, although we targeted individuals who identify as Christian in this study, the ways in which people practice Christianity have evolved over time and differ across contexts (and, of course, denominations) so a one-size-fits-all intervention for individuals who identify as Christian may be ineffective. As a result, the impact of the Christian-specific intervention utilized in this study—Centering Prayer—may not in fact amplify the effects of the general meditation practice that the active control group was instructed to complete. Prior studies have identified numerous modes and methods of religious coping, subsequently labelling them as ‘positive’ (i.e., congregational support, perceptions of a close and collaborative relationship with the divine) or ‘negative’ (i.e., viewing illness as divine punishment) religious coping, yet the effects of these practices remain largely dependent on the individual’s perceptions and the context in which they utilize these coping tools [46, 51]. Our choice to use Centering Prayer in this study, as noted previously, was based largely on a study by Ferguson and colleagues [19], which resulted in stress decreases and increases in the collaborative nature of participants’ relationships to God. However, Centering Prayer may not be perceived as important or effective by all Christians [17], and recent research has indicated the need to engage with and build upon the idiosyncratic practices of religious and/or spiritual individuals, rather than apply a singular intervention approach [70]. Furthermore, we did not directly assess perceived closeness to God from pre- to post-intervention, nor did we assess potential prior negative and positive religious coping skills and/or meditation practice more generally. On average, our sample reported moderate religiosity and the demographic make-up of our sample was highly skewed (73.65% White and 8.26% Hispanic). Future research should seek a more demographically representative sample and probe prior use of religious coping skills (both negative and positive) as well as mindfulness training more generally.

Third, as laid out in our discussion our Passive Control intervention may have been stronger than expected. In the case of this study, repeated reminders to be mindful and reflective of one’s day and the increased journaling response by the passive control group may indicate that the passive control was not, in fact, passive and could have produced some benefits in well-being. Future research should provide a weaker passive control group (i.e., an intervention shown to be ineffective) and seek to confirm compliance.

Additionally, it may be the case that existing constructs of well-being may not adequately capture the full spectrum of effects resulting from spiritual interventions. Aspects of spiritual health beyond flourishing may also be of value to some and could be worth studying. Following the example of Wachholtz and Pargament [67, 68], it may be useful to assess direct measures of pain tolerance or other factors that relate to daily functioning that can be physiologically assessed. Such physiological and direct health measures may also be possible to collect through new mobile technologies that assess physiological measures and/or health behaviors, including wearable devices, though we were unable to obtain usable data for these purposes in the present study. Obtaining direct measures and self-reports of the effects of these interventions is important given the differential effects that secular and religious-integrated intervention may have on different parts of one’s well-being.

Finally, we note that there is a widely acknowledged reproducibility crisis in science [6] and a sign of a healthy science is the publication of null results––unfortunately, psychology and psychiatry are among the worst offenders of failing to publish null results [18]. Accordingly, there have been recent calls to publish more null results [44], as we have done here.

## Conclusions

Our study comparing a religiously framed and a non-religiously framed meditation practice on well-being showed *no* between-group differences on our primary outcome measure of well-being. Each condition of our study increased in well-being from baseline to follow-up. We also found increases in positive affect and decreases in negative affect from baseline to follow-up across all conditions. Among the experimental group only (those assigned an explicitly religious intervention), we found a significantly elevated frequency of spiritual, mystical, and awe experiences. Our study was potentially limited by an inability to confirm compliance and a strong passive control condition. In addition to the instrumental value of increasing well-being, we might also consider investigating the role and value of experiences that could be considered religious, spiritual, mystical, or awe-inspiring regardless of their direct effects on general well-being. There is value in the scientific study of common practices (beyond mindfulness) from various world religions and worldviews [77]. Nonetheless, this study contributes to an existing—though highly mixed and relatively limited—body of literature indicating that integration of religion/spirituality into mindfulness interventions may not enhance the well-being effects of these practices.

### Supplementary Information

Supplementary Material 1.

Supplementary Material 2.

## Data Availability

Available on request.
