# Supplementary material for: Centering and flourishing: an online intervention study assessing the effects of a Christian contemplative practice on stress-reduction and human flourishing
Source: BMC Psychol. 2024 Jul 1;12:373. doi: 10.1186/s40359-024-01836-0 (PMC11218060; doi:10.1186/s40359-024-01836-0)
Supplement: Supplementary file 1 — Supplementary Material 1. [file 40359_2024_1836_MOESM1_ESM.docx]

**Additional File 1: SUPPLEMENT A**

**Additional File 1** contains Supplement A. All figures and tables in Supplement A are referenced in the main text as, for example, ‘Figure A1’ or ‘Table A4’.

**Supplementary Table A1**

*Summary of average total flourishing scores across all timepoints*

| **Timepoint** | **Group** | **Mean** | **SE** | **n** |
| --- | --- | --- | --- | --- |
| Baseline | Passive | 6.24 | 0.115 | 234 |
| One-day post |  | 6.64 | 0.121 | 234 |
| *One-week post* |  | 6.76 | 0.115 | 234 |
| One-month post |  | 6.79 | 0.140 | 170 |
| Baseline | Active | 6.41 | 0.125 | 234 |
| One-day post |  | 6.97 | 0.117 | 234 |
| *One-week post* |  | 7.04 | 0.119 | 234 |
| One-month post |  | 7.01 | 0.142 | 162 |
| Baseline | Experimental | 6.29 | 0.126 | 234 |
| One-day post |  | 6.88 | 0.121 | 234 |
| *One-week post* |  | 7.02 | 0.120 | 234 |
| One-month post |  | 6.97 | 0.155 | 154 |

*Note:* Total flourishing scores are summarized by group at each of the baseline and follow-up timepoints. Significant dropout at one-month post-intervention due to over-enrollment and resulting budget issues, requiring us to end the study early for many of our participants. N remained the same through one-week post-intervention based on our filtering criteria for data inclusion (must complete one-week post-intervention survey; 234 each group) but dropped at the one-month timepoint. SE represents standard error and n represents the sample size.

**Supplementary Table A2**

*Pairwise comparisons of multivariate regression models of total flourishing at all post-intervention timepoints.*

| **Outcome** | **Contrasts** | ***b*** | **SE** | ***p*** | **t-ratio** | ***d*** | **df** |
| --- | --- | --- | --- | --- | --- | --- | --- |
| *Total Flourishing,*  *One-week post* | Experimental – Active Control | 0.04 | 0.12 | 0.9448 | 0.32 | 0.03 | 693 |
|  | Experimental – Passive Control | 0.23 | 0.12 | 0.12 | 1.97 | 0.18 | 693 |
|  | Active Control – Passive Control | 0.19 | 0.12 | 0.2297 | 1.64 | 0.15 | 693 |
| Total Flourishing,  One-day post | Experimental – Active Control | -0.03 | 0.12 | 0.9703 | -0.23 | -0.02 | 693 |
|  | Experimental – Passive Control | 0.22 | 0.12 | 0.1414 | 1.89 | 0.18 | 693 |
|  | Active Control – Passive Control | 0.25 | 0.12 | 0.087 | 2.12 | 0.2 | 693 |
| Total Flourishing,  One-month post | Experimental – Active Control | 0.07 | 0.15 | 0.8865 | 0.47 | 0.05 | 477 |
|  | Experimental – Passive Control | 0.14 | 0.15 | 0.6171 | 0.94 | 0.1 | 477 |
|  | Active Control – Passive Control | 0.07 | 0.15 | 0.8873 | 0.47 | 0.05 | 477 |

*Note:* No significant between-group differences were found at in our primary outcome measure post-intervention. The primary outcome timepoint (one-week post-intervention) is italicized for emphasis. Regression models included all available demographic and related variables (race, ethnicity, sex, age, self-reported religiosity, as well as baseline scores, as covariates. No significant between-group differences in any of these covariates were found at baseline. For these pairwise comparisons, a Tukey family-wise adjustment was applied. SE refers to standard error and *d* refers to effects size (Cohen’s d).

**Supplementary Table A3**

*Results of paired t-tests on total flourishing at all post-intervention timepoints—one-week (primary), one-day, and one-month post-intervention—compared to baseline scores.*

| **Group** | **Flourishing Outcome** | **MD** | **95% CI** | **t ratio** | ***d*** | ***p*** |
| --- | --- | --- | --- | --- | --- | --- |
| Passive | *Total, one-week post* | 0.52 | [0.33, 0.7] | 5.52 | 0.29 | <0.001 |
|  | Total, one-day post | 0.38 | [0.21, 0.55] | 4.43 | 0.21 | <0.001 |
|  | Total, one-month post | 0.69 | [0.46, 0.93] | 5.81 | 0.38 | <0.001 |
| Active | *Total, one-week post* | 0.64 | [0.45, 0.82] | 6.86 | 0.34 | <0.001 |
|  | Total, one-day post | 0.56 | [0.39, 0.74] | 6.2 | 0.3 | <0.001 |
|  | Total, one-month post | 0.67 | [0.44, 0.89] | 5.88 | 0.35 | <0.001 |
| Experimental | *Total, one-week post* | 0.73 | [0.57, 0.9] | 8.76 | 0.39 | <0.001 |
|  | Total, one-day post | 0.59 | [0.41, 0.78] | 6.28 | 0.31 | <0.001 |
|  | Total, one-month post | 0.79 | [0.54, 1.03] | 6.39 | 0.4 | <0.001 |

*Note:* The primary outcome timepoint (one-week post-intervention) is italicized for emphasis. MD refers to mean difference, 95% CI refers to confidence interval, and *d* refers to effect size (Cohen’s d). Degrees of freedom (df) for each output is 233.

**Supplementary Table A4**

**Table 4 (A)**

*Fixed interaction effects of daily survey outcomes analyzed via mixed-effects model, with Passive Control as reference group.*

| **Outcome** | **Time by Group** | ***b (time x group)*** | **SE** | **t-ratio** | **df** | **p** |
| --- | --- | --- | --- | --- | --- | --- |
| Affect | Time: Active | 0.01 | 0.01 | 1.89 | 18217 | 0.0582 |
|  | Time: Experimental* | 0.01 | 0.01 | 2.16 | 18217 | 0.0304 |
| Sleep | Time: Active | 0 | 0 | -0.03 | 6043 | 0.9732 |
|  | Time: Experimental | 0.01 | 0 | 1.3 | 6043 | 0.1934 |
| Exercise | Time: Active | 0.01 | 0 | 1.37 | 5392 | 0.1694 |
|  | Time: Experimental | 0.01 | 0 | 1.74 | 5392 | 0.0821 |
| Social | Time: Active | 0.01 | 0.01 | 1.22 | 5372 | 0.2227 |
|  | Time: Experimental | 0.01 | 0.01 | 1.05 | 5372 | 0.2957 |

**Table 4 (B)**

*Simple slopes (fixed effect) of time on linear mixed-effects of daily survey outcome variable models, by group, over the course of the intervention period.*

| **Variable** | **Group** | ***b* (time)** | **SE** | **t-ratio** | **df** | **p** |
| --- | --- | --- | --- | --- | --- | --- |
| Affect | Passive | 0.007 | 0 | 1.857 | 18217 | 0.063 |
|  | Active*** | 0.016 | 0 | 4.536 | 18217 | <0.001 |
|  | Experimental*** | 0.018 | 0 | 4.921 | 18217 | <0.001 |
| Sleep | Passive | 0.006 | 0 | 1.864 | 6043 | 0.062 |
|  | Active | 0.005 | 0 | 1.819 | 6043 | 0.069 |
|  | Experimental*** | 0.011 | 0 | 3.71 | 6043 | <0.001 |
| Exercise | Passive** | 0.009 | 0 | 2.704 | 5392 | 0.007 |
|  | Active*** | 0.015 | 0 | 4.648 | 5392 | <0.001 |
|  | Experimental*** | 0.017 | 0 | 5.171 | 5392 | <0.001 |
| Social | Passive | 0.002 | 0 | 0.475 | 5372 | 0.635 |
|  | Active* | 0.009 | 0 | 2.199 | 5372 | 0.028 |
|  | Experimental | 0.008 | 0 | 1.953 | 5372 | 0.051 |

*Note: b* refers to fixed effect of time by group (A) and simple slopes of time per group, SE refers to standard error, and df refers to degrees of freedom. Significance is indicated in the “Group by time” (A) or “Group” (B) column (*p<0.05, **p<0.01, ***p<0.001). **(A)** Time by group interactions reflect the fixed effect of behavioral intervention (group); p-values reflect differences between groups in the linear mixed-effects of daily survey outcomes with Passive Control as the reference group in each case. **(B)** Simple slopes of time (b), by group, are essentially the contribution of the fixed effect of time to the linear models of the daily survey outcome, by group. These values are compared to a zero slope, indicating whether participation in the intervention resulted in a positive increase in these health behaviors (compared to no effect, zero slope).

**Supplementary Figure A1**

*Self-reported daily survey outcomes, dichotomized over intervention period (Days 1-14 and Days 15-28, separately). (A) Affect balance; (B) Sleep, exercise, and social engagement over the intervention, dichotomized as a function of group (behavioral intervention).*

| (A)    **  *** | (B)    **  * *** ***  * |
| --- | --- |

*Note:* Horizontal bars and vertical error bars within violin plots signify mean values and 95% CIs, respectively. Significance over time, by group, is indicated via asterisks over the individual group- and outcome-specific violin plots; *p<0.05; **p<0.01, ***p<0.001.

**Supplementary Table A5**

*Within-group differences in affect balance and health behaviors over dichotomized (Days 1-14 vs. Days 15-28) intervention period, by group.*

| **Group** | **Outcome** | ***p*** | **MD** | **95% CI** | **t ratio** | **Cohen's d** |
| --- | --- | --- | --- | --- | --- | --- |
| Passive | Affect Balance | 0.064 | 0.1 | [-0.01, 0.2] | 1.86 | 0.06 |
|  | Social | 0.693 | 0.02 | [-0.09, 0.13] | 0.39 | 0.02 |
|  | Exercise* | 0.01 | 0.13 | [0.03, 0.22] | 2.59 | 0.11 |
|  | Sleep | 0.138 | 0.08 | [-0.02, 0.18] | 1.49 | 0.07 |
| Active | Affect Balance** | 0.001 | 0.2 | [0.09, 0.32] | 3.46 | 0.13 |
|  | Social* | 0.03 | 0.13 | [0.01, 0.25] | 2.18 | 0.11 |
|  | Exercise*** | <0.001 | 0.21 | [0.12, 0.3] | 4.57 | 0.19 |
|  | Sleep | 0.519 | 0.03 | [-0.06, 0.12] | 0.65 | 0.03 |
| Experimental | Affect Balance*** | <0.001 | 0.22 | [0.1, 0.34] | 3.54 | 0.13 |
|  | Social | 0.127 | 0.09 | [-0.03, 0.21] | 1.53 | 0.08 |
|  | Exercise*** | <0.001 | 0.24 | [0.13, 0.34] | 4.48 | 0.2 |
|  | Sleep** | 0.006 | 0.16 | [0.04, 0.27] | 2.77 | 0.14 |

*Note:* Paired t-test and effect size (Cohen’s d) of dichotomized daily outcomes, comparing averaged values over Days 15-28 to Days 1-14, by group. CI refers confidence interval and MD refers to mean difference values. Degrees of freedom (df) for each output is 233. Significance of results is indicated in the “Outcome” column; *p<0.05; **p<0.01, ***p<0.001.

**Supplementary Table A6**

*Pairwise comparisons of multivariate regression models of exploratory outcomes (ESAT, AWE-S, MEQ, DSES).*

| **Outcome** | **Contrasts** | ***b*** | **SE** | ***p*** | **t ratio** | ***d*** | **df** |
| --- | --- | --- | --- | --- | --- | --- | --- |
| ESAT Negative | Experimental - Active | 0.08 | 0.06 | 0.466 | 1.18 | 0.11 | 693 |
|  | Experimental - Passive | -0.11 | 0.06 | 0.2078 | -1.69 | -0.16 | 693 |
|  | Active – Passive* | -0.18 | 0.06 | 0.0118 | -2.87 | -0.27 | 693 |
| ESAT Positive | Experimental - Active | 0.01 | 0.06 | 0.9725 | 0.23 | 0.02 | 693 |
|  | Experimental - Passive | 0.13 | 0.06 | 0.0696 | 2.21 | 0.2 | 693 |
|  | Active – Passive | 0.11 | 0.06 | 0.1189 | 1.98 | 0.18 | 693 |
| AWE Total | Experimental - Active | 0.19 | 0.09 | 0.0628 | 2.26 | 0.21 | 693 |
|  | Experimental – Passive | 0.18 | 0.09 | 0.0898 | 2.1 | 0.2 | 693 |
|  | Active - Passive | -0.01 | 0.09 | 0.9853 | -0.16 | -0.02 | 693 |
| MEQ Total | Experimental – Active | 0.2 | 0.09 | 0.076 | 2.18 | 0.2 | 694 |
|  | Experimental – Passive*** | 0.35 | 0.09 | 0.0003 | 3.95 | 0.37 | 694 |
|  | Active - Passive | 0.16 | 0.09 | 0.1873 | 1.75 | 0.16 | 694 |
| DSES Total | Experimental - Active | 2.95 | 1.56 | 0.1425 | 1.89 | 0.18 | 694 |
|  | Experimental – Passive*** | 6.46 | 1.55 | 0.0001 | 4.16 | 0.39 | 694 |
|  | Active – Passive | 3.51 | 1.56 | 0.0635 | 2.25 | 0.21 | 694 |
| DSES Total (one-week) | Experimental - Active | 3.01 | 1.56 | 0.1307 | 1.93 | 0.18 | 694 |
|  | Experimental – Passive** | 5.04 | 1.55 | 0.0033 | 3.26 | 0.3 | 694 |
|  | Active - Passive | 2.03 | 1.55 | 0.3896 | 1.31 | 0.12 | 694 |
| DSES Total (one-month) | Experimental - Active | 3.83 | 1.85 | 0.0958 | 2.08 | 0.23 | 478 |
|  | Experimental – Passive* | 5.95 | 1.82 | 0.0033 | 3.27 | 0.36 | 478 |
|  | Active - Passive | 2.12 | 1.8 | 0.4693 | 1.17 | 0.13 | 478 |

*Note:* Exploratory outcome variables were collected at one-day post-intervention. For pairwise comparisons, a Tukey family-wise adjustment was applied. Covariates for regressions included all available demographic and related variables, as well as baseline scores when available (no significant differences between-groups at baseline). Significance of post-intervention pairwise comparison is noted in the “Contrasts” (referring to which groups are being compared via the pairwise comparisons) column, *p<0.05, **p<0.01, ***p<0.001. SE refers to standard error and *d* refers to effects size (Cohen’s *d*).

**Supplementary Table A7**

*Paired t-tests of exploratory outcomes with available baseline scores (ESAT and AWE-S).*

| **Group** | **Outcome** | ***p*** | **MD** | **95% CI** | **t ratio** | ***d*** |
| --- | --- | --- | --- | --- | --- | --- |
| Passive | AWE Total | 0.429 | 0.06 | [-0.08, 0.2] | 0.79 | 0.05 |
|  | ESAT Positive | <0.001 | 0.2 | [0.12, 0.29] | 4.66 | 0.24 |
|  | ESAT Negative | <0.001 | -0.2 | [-0.3, -0.1] | -4.02 | -0.21 |
|  | ESAT Positive *(w)* | <0.001 | 0.25 | [0.15, 0.34] | 5.28 | 0.29 |
|  | ESAT Negative *(w)* | <0.001 | -0.34 | [-0.44, -0.24] | -6.48 | -0.36 |
|  | ESAT Positive *(m)* | <0.001 | 0.29 | [0.17, 0.41] | 4.86 | 0.36 |
|  | ESAT Negative *(m)* | <0.001 | -0.38 | [-0.52, -0.25] | -5.61 | -0.39 |
| Active | AWE Total | 0.93 | -0.01 | [-0.15, 0.14] | -0.09 | -0.01 |
|  | ESAT Positive | <0.001 | 0.29 | [0.2, 0.37] | 6.63 | 0.35 |
|  | ESAT Negative | <0.001 | -0.34 | [-0.43, -0.25] | -7.47 | -0.36 |
|  | ESAT Positive *(w)* | <0.001 | 0.23 | [0.15, 0.31] | 5.5 | 0.28 |
|  | ESAT Negative *(w)* | <0.001 | -0.34 | [-0.44, -0.25] | -7.18 | -0.36 |
|  | ESAT Positive *(m)* | <0.001 | 0.24 | [0.14, 0.35] | 4.61 | 0.29 |
|  | ESAT Negative *(m)* | <0.001 | -0.37 | [-0.51, -0.24] | -5.62 | -0.37 |
| Experimental | AWE Total | 0.01 | 0.16 | [0.04, 0.29] | 2.58 | 0.17 |
|  | ESAT Positive | <0.001 | 0.32 | [0.23, 0.4] | 7.41 | 0.38 |
|  | ESAT Negative | <0.001 | -0.31 | [-0.41, -0.21] | -6.09 | -0.31 |
|  | ESAT Positive *(w)* | <0.001 | 0.31 | [0.22, 0.41] | 6.68 | 0.37 |
|  | ESAT Negative *(w)* | <0.001 | -0.38 | [-0.47, -0.28] | -7.48 | -0.38 |
|  | ESAT Positive *(m)* | <0.001 | 0.38 | [0.27, 0.49] | 6.77 | 0.44 |
|  | ESAT Negative *(m)* | <0.001 | -0.45 | [-0.58, -0.31] | -6.49 | -0.44 |

*Note:* Exploratory outcome variables at one-day follow-up compared to available baseline scores (ESAT and AWE-S) were analyzed via paired t-tests. ESAT factor outcomes at one-week and one-month post-intervention were similarly analyzed to determine if significant differences were sustained. Outcome reflects one-day follow-up unless indicated otherwise; *(w)* reflects one-week and *(m)* reflects one-month post-intervention outcomes. MD refers to mean difference error and *d* refers to effects size (Cohen’s d).

***Pre-cursor Centering Words Survey: Determining “Neutral” and “Sacred” Words***

Participants of this sub-study were recruited through an online invitation on Amazon’s Mechanical Turk (M-Turk). Inclusion criteria mirrored the conditions for the main Centering Study: All participants were 18+, identified as Christian and rated above neutral on a self-report question of religiosity. A list of 40 potential “sacred” and “neutral” words were selected from Linguistic Inquiry and Word Count (LIWC; Tausczik & Pennebaker, 2010) dictionaries by the research team and presented to participants in a randomized order (using Qualtrics’ simple display randomizer). Participants were asked to rate each word on a scale of “Neutral” (0) to “Sacred” (5). Self-identifying Christians (N=217), approximately 75% of whom were between 25-44 years-old and 89% of whom identified as either “moderately religious” or “very religious,” responded to the survey and the mean rating of each word was calculated. The top 10 most “neutral” and “sacred” words, respectively, were determined based on their mean values and are displayed in the Table 2.

**Supplementary Table A8**

*Results of pre-cursor survey, the Neutral and Sacred Words Survey (N=217)*

| **Top-ten Sacred Words** | | **Top-ten Neutral Words** | |
| --- | --- | --- | --- |
| **Word** | **Mean (SD)** | **Word** | **Mean (SD)** |
| God | 2.44 (0.91) | Ball | 0.85 (1.11) |
| Jesus Christ | 2.37 (0.99) | Ticket | 0.85 (1.09) |
| Sacred | 2.32 (0.96) | Furniture | 0.85 (1.07) |
| Heaven | 2.32 (0.95) | Yard | 0.85 (1.06) |
| Bible | 2.29 (0.97) | Pillow | 0.85 (1.11) |
| Holy | 2.25 (0.92) | Bed | 0.84 (1.04) |
| Angel | 2.17 (0.94) | Eleven | 0.83 (1.07) |
| Amen | 2.13 (0.97) | Coffee | 0.82 (1.06) |
| Bless | 2.10 (0.94) | Bike | 0.79 (1.05) |
| Soul | 2.09 (0.93) | Soap | 0.76 (1.04) |

*Note.* A list of 40 potential “sacred” and “neutral” words were selected from LIWC dictionaries and presented to survey participants in a randomized order). Participants (N=217) were asked to rate each word on a scale of “Neutral” (0) to “Sacred” (5) and the mean reported “sacredness” of each word was calculated. The top 10 most “neutral” and “sacred” words, respectively, were determined based on their degree of reported “sacredness” and are displayed above. Mean values reflect the degree of reported sacredness.

These top-ten lists were included in the condition-appropriate instructions for the experimental and active control groups in the main Centering Study. These instructions are included in the table below (see ***Table 2***). At baseline, participants in the experimental and active control groups were asked to select one of the ten words provided to them. They were reminded of this selection throughout their participation in the study.

**Supplementary Figure A2**

*Consort diagram of participant inclusion in data analysis sample*

**
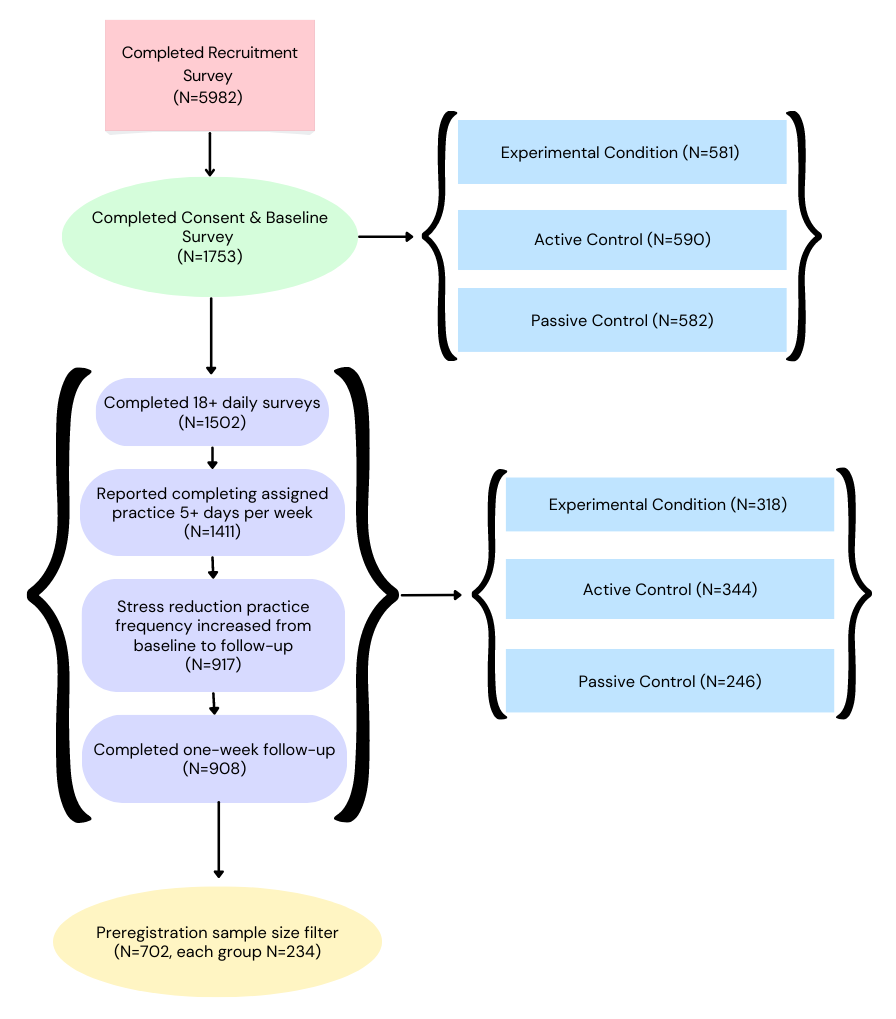
**

*Note.* Inclusion in data analysis was based on pre-registered exclusion criteria. The first 234 participants within each group (Experimental, Active Control, Passive Control) to complete the study and meet all inclusion criteria were selected for inclusion in preregistered sample analyses. All participants who met inclusion criteria for analysis were included in the full sample analyses (Supplementary Material B).
