# Supplementary material for: Centering and flourishing: an online intervention study assessing the effects of a Christian contemplative practice on stress-reduction and human flourishing
Source: BMC Psychol. 2024 Jul 1;12:373. doi: 10.1186/s40359-024-01836-0 (PMC11218060; doi:10.1186/s40359-024-01836-0)
Supplement: Supplementary file 2 — Supplementary Material 2. [file 40359_2024_1836_MOESM2_ESM.docx]

**SUPPLEMENT B (Full Sample Supplement)**

**Additional File 2** contains Supplement B. Findings from the full sample, rather than the preregistered sample size, are included in Supplement B. They are named as, for example, ‘Figure B2’ or ‘Table B1’.

***Limitation of the Full Sample Results*:** The different sample sizes and retention rates observed across groups are an additional limitation within the full sample supplement and analyses.

**Full Sample Supplementary Table B1**

*Demographic Information for Full Sample (N=908)*

| **Variable** | **Average Value** |
| --- | --- |
| Age (SD) | 44.88 (24.34) |
| Extent Religious (SD) | 3.10 (0.69) |
| Female (% female) | 609 (67.07%) |
| Hispanic (% Hispanic) | 80 (8.81%) |
| Race (count (% of sample)) | |
| Black | 181 (19.93%) |
| White | 667 (73.46%) |
| Multi-racial/Other | 53 (5.84%) |

**Note:* Religiosity was measured on a scale from 0-4 (“Not religious” to “Very religious”), with higher scores indicating a higher extent to which a participant identified as religious and a value of three most closely aligning with the response, “Moderately religious” on this scale. SD = Standard Deviation. Individuals were randomized to an assigned behavioral intervention and no significant differences in demographics were found between groups; Passive Control (N=246), Active Control (N=344), Experimental (N=318).

**Full Sample Supplementary Figure B1**

*Total flourishing scores plotted over time.*

*Note.* Pairwise comparisons of total flourishing scores were conducted to assess between-group differences; no significant between-group differences in total flourishing scores were found (ns = not significant). Paired t-tests on total flourishing scores were conducted between all post-intervention timepoints and baseline scores by group. Paired t-tests revealed significant increases in total flourishing for all groups. The triple bars of each timepoint comparison signify that the asterisk (***p<0.001) reflects results of paired t-test analyses conducted within all three groups. Range of total flourishing score values is 0-8. Error bars represent standard error.

**Full Sample Supplementary Table B2**

*Summary of average total flourishing scores across all timepoints for full sample*

| **Timepoint** | **Group** | **Mean** | **SE** | **n** |
| --- | --- | --- | --- | --- |
| Baseline | Passive | 6.20 | 0.11 | 246 |
| One-day post |  | 6.61 | 0.12 | 246 |
| *One-week post* |  | 6.76 | 0.11 | 246 |
| One-month post |  | 6.78 | 0.14 | 179 |
| Baseline | Active | 6.45 | 0.10 | 344 |
| One-day post |  | 7.03 | 0.10 | 344 |
| *One-week post* |  | 7.09 | 0.10 | 344 |
| One-month post |  | 7.03 | 0.12 | 240 |
| Baseline | Experimental | 6.34 | 0.11 | 318 |
| One-day post |  | 6.87 | 0.11 | 318 |
| *One-week post* |  | 7.02 | 0.10 | 318 |
| One-month post |  | 6.98 | 0.13 | 214 |

**Note:* Total flourishing scores are summarized by group at each of the baseline and follow-up timepoints. Significant dropout at one-month post-intervention due to over-enrollment and resulting budget issues, requiring us to end the study early for many of our participants. N remained the same through one-week post-intervention based on our filtering criteria for data inclusion but dropped at the one-month timepoint: Experimental (N=179), Active (N=240), and Control (N=214). SE represents standard error and n represents the sample size.

**Full Sample Supplementary Table B3**

*Pairwise comparisons of multivariate regression models of total flourishing at all post-intervention timepoints in the full sample*

| **Outcome** | **Contrasts** | ***b*** | **SE** | ***p*** | **t-ratio** | ***d*** | **df** |
| --- | --- | --- | --- | --- | --- | --- | --- |
| *Total Flourishing,*  *One-week post* | Experimental – Active Control | 0 | 0.1 | 0.9992 | -0.04 | 0 | 899 |
|  | Experimental – Passive Control | 0.16 | 0.11 | 0.2954 | 1.49 | 0.13 | 899 |
|  | Active Control – Passive Control | 0.16 | 0.1 | 0.2688 | 1.55 | 0.13 | 899 |
| Total Flourishing,  One-day post | Experimental – Active Control | -0.09 | 0.1 | 0.6138 | -0.94 | -0.07 | 899 |
|  | Experimental – Passive Control | 0.15 | 0.11 | 0.3408 | 1.4 | 0.12 | 899 |
|  | Active Control – Passive Control | 0.24 | 0.11 | 0.0563 | 2.3 | 0.19 | 899 |
| Total Flourishing,  One-month post | Experimental – Active Control | 0.01 | 0.13 | 0.9974 | 0.07 | 0.01 | 624 |
|  | Experimental – Passive Control | 0.07 | 0.14 | 0.8629 | 0.52 | 0.05 | 624 |
|  | Active Control – Passive Control | 0.06 | 0.13 | 0.8883 | 0.46 | 0.05 | 624 |

*Note:* The primary outcome timepoint (one-week post-intervention) is italicized for emphasis. Regression models included demographic factors that were significantly correlated with the outcome and baseline score. SE refers to standard error and *d* refers to effects size (Cohen’s d). No significant between-group differences were found.

**Full Sample Supplementary Table B4**

*Results of paired t-tests on total flourishing at all post-intervention timepoints—one-week (primary), one-day, and one-month post-intervention compared to baseline scores—in the full sample.*

| **Group** | **Flourishing Outcome** | **MD** | **95% CI** | **t ratio** | ***d*** | ***p*** |
| --- | --- | --- | --- | --- | --- | --- |
| Passive | *Total, one-week post* | 0.56 | [0.38, 0.74] | 6.02 | 0.32 | <0.001 |
|  | Total, one-day post | 0.41 | [0.24, 0.58] | 4.84 | 0.23 | <0.001 |
|  | Total, one-month post | 0.75 | [0.52, 0.99] | 6.35 | 0.41 | <0.001 |
| Active | *Total, one-week post* | 0.64 | [0.49, 0.79] | 8.51 | 0.34 | <0.001 |
|  | Total, one-day post | 0.58 | [0.44, 0.72] | 7.92 | 0.31 | <0.001 |
|  | Total, one-month post | 0.71 | [0.53, 0.89] | 7.73 | 0.37 | <0.001 |
| Experimental | *Total, one-week post* | 0.68 | [0.54, 0.81] | 9.79 | 0.36 | <0.001 |
|  | Total, one-day post | 0.52 | [0.37, 0.67] | 6.89 | 0.28 | <0.001 |
|  | Total, one-month post | 0.74 | [0.55, 0.94] | 7.42 | 0.38 | <0.001 |

*Note:* The primary outcome timepoint (one-week post-intervention) is italicized for emphasis. MD refers to mean difference, 95% CI refers to confidence interval, and *d* refers to effects size (Cohen’s d).

**Full Sample Supplementary Figure B2**

*Linear mixed-effects model of self-reported daily affect balance, sleep, exercise, and social engagement over the intervention period, by group.*

| (A)  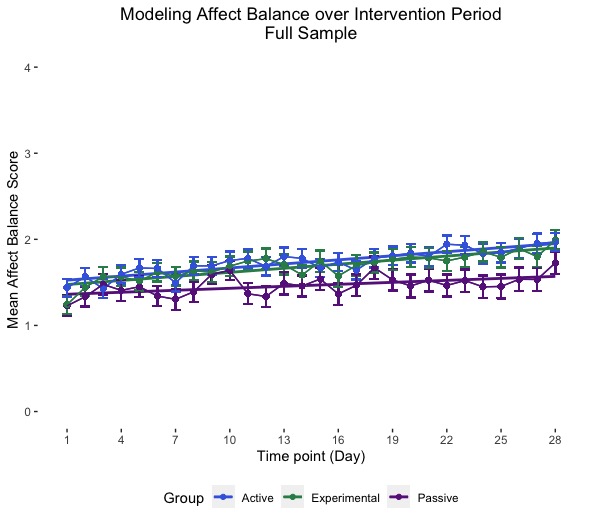 | (B)  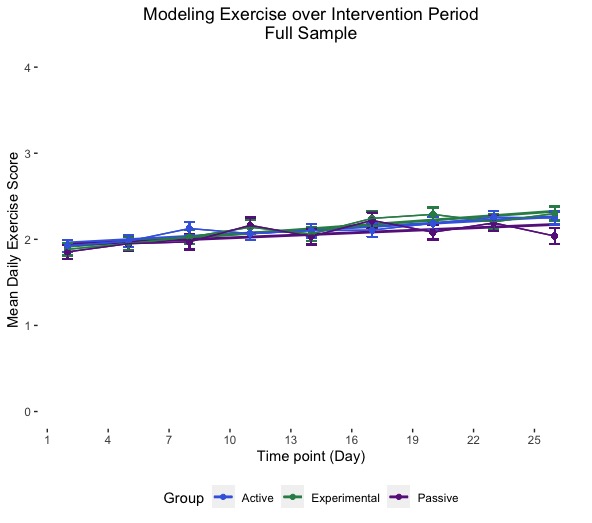 |
| --- | --- |
| (C)  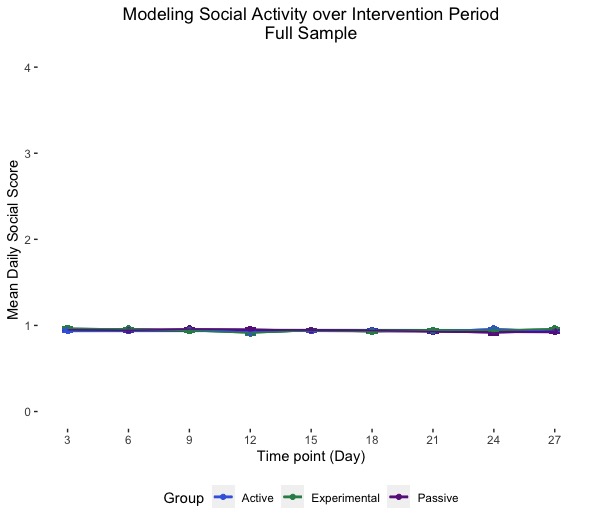 | (D)  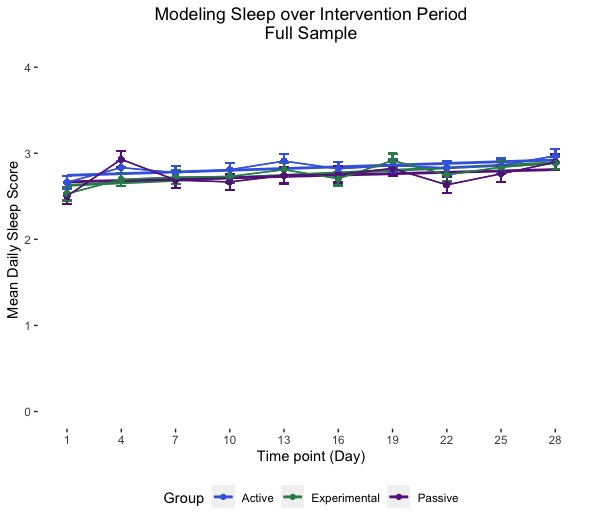 |

*Note:* Mean values of self-reported daily survey outcomes by day and group are plotted, overlayed with linear mixed-effects model. Error bars represent standard error. (**A**) Comparisons of the affect balance models revealed no significant differences between groups. Daily affect balance scores range from -4 to 4. (**B**, **C**, **D**) Linear mixed-effects model of self-reported sleep, exercise, and social engagement over the intervention period, by group. No significant differences in sleep, exercise, or social engagement were found between group. Mean daily sleep, exercise, and social engagement scores range from 0 to 4.

**Full Sample Supplementary Table B5**

**Table 5 (A)**

*Fixed interaction effects of daily survey outcomes analyzed via mixed-effects model, with Passive Control as reference group.*

| **Outcome** | **Time by Group** | ***b (time x group)*** | **SE** | **t-ratio** | **df** | **p** |
| --- | --- | --- | --- | --- | --- | --- |
| Affect | Time: Active | 0.01 | 0 | 1.88 | 23587 | 0.0605 |
|  | Time: Experimental | 0.01 | 0 | 1.8 | 23587 | 0.0714 |
| Sleep | Time: Active | 0 | 0 | 0.12 | 7827 | 0.9048 |
|  | Time: Experimental | 0 | 0 | 0.98 | 7827 | 0.325 |
| Exercise | Time: Active | 0 | 0 | 0.88 | 6980 | 0.3794 |
|  | Time: Experimental | 0.01 | 0 | 1.54 | 6980 | 0.1236 |
| Social | Time: Active | 0 | 0.01 | 0.78 | 6958 | 0.4349 |
|  | Time: Experimental | 0 | 0.01 | 0.38 | 6958 | 0.7072 |

**Table 5 (B)**

*Simple slopes (fixed effect) of time on linear mixed-effects of daily survey outcome variable models, by group, over the course of the intervention period.*

| **Variable** | **Group** | ***b* (time)** | **SE** | **t-ratio** | **df** | **p** |
| --- | --- | --- | --- | --- | --- | --- |
| Affect | Passive* | 0.008 | 0 | 2.141 | 23587 | 0.032 |
|  | Active*** | 0.016 | 0 | 5.436 | 23587 | <0.001 |
|  | Experimental*** | 0.016 | 0 | 5.169 | 23587 | <0.001 |
| Sleep | Passive* | 0.006 | 0 | 2.124 | 7827 | 0.034 |
|  | Active** | 0.007 | 0 | 2.696 | 7827 | 0.007 |
|  | Experimental*** | 0.01 | 0 | 3.915 | 7827 | <0.001 |
| Exercise | Passive** | 0.009 | 0 | 2.899 | 6980 | 0.004 |
|  | Active*** | 0.013 | 0 | 4.79 | 6980 | <0.001 |
|  | Experimental*** | 0.016 | 0 | 5.631 | 6980 | <0.001 |
| Social | Passive | 0.002 | 0 | 0.49 | 6958 | 0.624 |
|  | Active | 0.006 | 0 | 1.788 | 6958 | 0.074 |
|  | Experimental | 0.004 | 0 | 1.128 | 6958 | 0.259 |

*Note: b* refers to fixed effect of time by group (A) and simple slopes of time per group, SE refers to standard error, and df refers to degrees of freedom. Significance is indicated in the “Group by time” (A) or “Group” (B) column (*p<0.05, **p<0.01, ***p<0.001). **(A)** Time by group interactions reflect the fixed effect of behavioral intervention (group); p-values reflect differences between groups in the linear mixed-effects of daily survey outcomes with Passive Control as the reference group in each case. **(B)** Simple slopes of time (b), by group, are essentially the contribution of the fixed effect of time to the linear models of the daily survey outcome, by group. These values are compared to a zero slope, indicating whether participation in the intervention resulted in a positive increase in these health behaviors (compared to no effect, zero slope).

**Full Sample Supplementary Figure B3**

*Self-reported daily survey outcomes, dichotomized over intervention period (Days 1-14 and Days 15-28, separately). (A) Affect balance; (B) Sleep, exercise, and social engagement over the intervention, dichotomized as a function of group (behavioral intervention).*

| (A)   | (B)   |
| --- | --- |

Self-reported (A) affect balance and (B) sleep, exercise, and social engagement over the intervention, dichotomized (Days 1-14 and Days 15-28, separately) as a function of group (behavioral intervention). Horizontal bars and error bars within violin plots are means and 95% CIs, respectively. Significance over time, by group, is indicated via asterisks over the individual group- and outcome-specific violin plots. No significant between-group differences were found, and within-group difference calculation results are in the table below.

**Full Sample Supplementary Table B6**

*Within-group differences in affect balance and health behaviors over dichotomized (Days 1-14 vs. Days 15-28) intervention period, by group.*

| **Group** | **Outcome** | ***p*** | **MD** | **95% CI** | **t ratio** | **Cohen's d** |
| --- | --- | --- | --- | --- | --- | --- |
| Passive | Affect Balance* | 0.036 | 0.1 | [0.01, 0.2] | 2.11 | 0.07 |
|  | Social | 0.660 | 0.02 | [-0.08, 0.13] | 0.44 | 0.02 |
|  | Exercise** | 0.006 | 0.13 | [0.04, 0.23] | 2.76 | 0.12 |
|  | Sleep | 0.099 | 0.08 | [-0.02, 0.18] | 1.65 | 0.07 |
| Active | Affect Balance*** | <0.001 | 0.19 | [0.1, 0.29] | 3.97 | 0.13 |
|  | Social | 0.052 | 0.09 | [0, 0.19] | 1.95 | 0.08 |
|  | Exercise*** | <0.001 | 0.17 | [0.09, 0.24] | 4.17 | 0.15 |
|  | Sleep | 0.107 | 0.06 | [-0.01, 0.14] | 1.62 | 0.05 |
| Experimental | Affect Balance*** | <0.001 | 0.2 | [0.1, 0.3] | 3.89 | 0.13 |
|  | Social | 0.409 | 0.04 | [-0.06, 0.15] | 0.83 | 0.04 |
|  | Exercise*** | <0.001 | 0.24 | [0.15, 0.33] | 5.29 | 0.2 |
|  | Sleep** | 0.004 | 0.13 | [0.04, 0.22] | 2.89 | 0.12 |

*Note:* Paired t-test and effect size (Cohen’s d) of dichotomized daily outcomes, comparing averaged values over Days 15-28 to Days 1-14, by group. CI refers confidence interval and MD refers to mean difference values. Significance of results is indicated in the “Outcome” column; *p<0.05; **p<0.01, ***p<0.001.

**Full Sample Figure B4**

*ESAT factor (positive and negative) and AWE-S total scores at baseline and one-day post-intervention*

*Note:* Significant paired t-test results (horizontal, on the top of the plots) and pairwise comparisons (on the right side of the plots) are marked by an asterisk (**p*<0.05, ***p*<0.01, ****p*<0.001). The triple horizontal bars over the ESAT factor score plots signify that the asterisk reflects results of paired t-test analyses conducted within all three groups. Pairwise comparisons revealed significant differences between the Active and Passive conditions on the ESAT Negative Factor Scores at one-day post-intervention. Similar pairwise comparisons revealed significant AWE-S score differences between the Experimental and Active conditions at one-day post-intervention. Error bars represent standard error.

**Full Sample Supplementary Figure B5**

*Mean (A) DSES and (B) MEQ-30 outcomes at one-day post-intervention.*

*Note:* Mean values by group are plotted and significant results of pairwise comparisons of multivariate regressions are represented by asterisks, ** p<0.01; ***p<0.001. Error bars represent standard error.


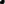

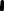

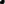

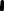


**Full Sample Supplementary Table B7**

*Pairwise comparisons of multivariate regression models of exploratory outcomes (ESAT, AWE-S, MEQ, DSES)*

| **Outcome** | **Contrasts** | ***b*** | **SE** | **p** | **t ratio** | ***d*** | **df** |
| --- | --- | --- | --- | --- | --- | --- | --- |
| ESAT Negative | Experimental - Active | 0.07 | 0.05 | 0.4621 | 1.19 | 0.09 | 899 |
|  | Experimental - Passive | -0.09 | 0.06 | 0.2484 | -1.59 | -0.14 | 899 |
|  | Active – Passive* | -0.16 | 0.06 | 0.018 | -2.73 | -0.23 | 899 |
| ESAT Positive | Experimental - Active | -0.01 | 0.05 | 0.9938 | -0.11 | -0.01 | 899 |
|  | Experimental – Passive | 0.1 | 0.05 | 0.1244 | 1.95 | 0.17 | 899 |
|  | Active - Passive | 0.11 | 0.05 | 0.0948 | 2.08 | 0.17 | 899 |
| AWE Total | Experimental – Active* | 0.17 | 0.07 | 0.0468 | 2.37 | 0.19 | 899 |
|  | Experimental – Passive | 0.13 | 0.08 | 0.2103 | 1.69 | 0.14 | 899 |
|  | Active – Passive | -0.04 | 0.08 | 0.8706 | -0.5 | -0.04 | 899 |
| MEQ Total | Experimental – Active | 0.15 | 0.08 | 0.135 | 1.91 | 0.15 | 900 |
|  | Experimental – Passive** | 0.29 | 0.08 | 0.0017 | 3.46 | 0.29 | 900 |
|  | Active – Passive | 0.14 | 0.08 | 0.1983 | 1.72 | 0.14 | 900 |
| DSES Total | Experimental – Active | 2.2 | 1.3 | 0.2065 | 1.7 | 0.13 | 900 |
|  | Experimental – Passive*** | 5.44 | 1.41 | 0.0004 | 3.86 | 0.33 | 900 |
|  | Active – Passive | 3.24 | 1.39 | 0.0514 | 2.34 | 0.2 | 900 |
| DSES Total (one-week) | Experimental – Active | 1.98 | 1.3 | 0.2788 | 1.53 | 0.12 | 900 |
|  | Experimental – Passive** | 4.17 | 1.41 | 0.009 | 2.96 | 0.25 | 900 |
|  | Active – Passive | 2.19 | 1.39 | 0.2575 | 1.57 | 0.13 | 900 |
| DSES Total (one-month) | Experimental – Active | 2.85 | 1.56 | 0.1602 | 1.83 | 0.17 | 625 |
|  | Experimental – Passive** | 5.1 | 1.67 | 0.0067 | 3.05 | 0.31 | 625 |
|  | Active – Passive | 2.25 | 1.63 | 0.3536 | 1.38 | 0.14 | 625 |

*Note:* Exploratory outcome variables were collected at one-day post-intervention. Significance of results is noted in the “Contrasts” (referring to which groups are being compared via the pairwise comparisons) column, *p<0.05, **p<0.01, ***p<0.001. SE refers to standard error and *d* refers to effects size (Cohen’s d).

**Full Sample Supplementary Table B8**

*Paired t-tests of exploratory outcomes with available baseline scores (ESAT and AWE-S).*

| **Group** | **Outcome** | ***p*** | **MD** | **95% CI** | **t ratio** | ***d*** |
| --- | --- | --- | --- | --- | --- | --- |
| Passive | AWE Total | 0.4165 | 0.06 | [-0.08, 0.19] | 0.81 | 0.05 |
|  | ESAT Positive | <0.001 | 0.22 | [0.14, 0.31] | 5.24 | 0.27 |
|  | ESAT Negative | <0.001 | -0.23 | [-0.33, -0.13] | -4.68 | -0.24 |
|  | ESAT Positive *(w)* | <0.001 | 0.27 | [0.18, 0.36] | 5.85 | 0.32 |
|  | ESAT Negative *(w)* | <0.001 | -0.37 | [-0.47, -0.27] | -7.15 | -0.4 |
|  | ESAT Positive *(m)* | <0.001 | 0.3 | [0.19, 0.42] | 5.2 | 0.37 |
|  | ESAT Negative *(m)* | <0.001 | -0.4 | [-0.53, -0.26] | -5.93 | -0.41 |
| Active | AWE Total | 0.6666 | -0.03 | [-0.14, 0.09] | -0.43 | -0.02 |
|  | ESAT Positive | <0.001 | 0.29 | [0.22, 0.36] | 8.05 | 0.35 |
|  | ESAT Negative | <0.001 | -0.34 | [-0.42, -0.26] | -8.25 | -0.35 |
|  | ESAT Positive *(w)* | <0.001 | 0.26 | [0.19, 0.34] | 7.36 | 0.32 |
|  | ESAT Negative *(w)* | <0.001 | -0.37 | [-0.45, -0.29] | -8.9 | -0.39 |
|  | ESAT Positive *(m)* | <0.001 | 0.28 | [0.19, 0.37] | 6.29 | 0.34 |
|  | ESAT Negative *(m)* | <0.001 | -0.4 | [-0.51, -0.28] | -7.01 | -0.4 |
| Experimental | AWE Total | 0.0986 | 0.1 | [-0.02, 0.21] | 1.66 | 0.1 |
|  | ESAT Positive | <0.001 | 0.31 | [0.24, 0.39] | 8.4 | 0.38 |
|  | ESAT Negative | <0.001 | -0.32 | [-0.41, -0.24] | -7.29 | -0.33 |
|  | ESAT Positive *(w)* | <0.001 | 0.32 | [0.24, 0.4] | 7.83 | 0.38 |
|  | ESAT Negative *(w)* | <0.001 | -0.38 | [-0.46, -0.29] | -8.53 | -0.38 |
|  | ESAT Positive *(m)* | <0.001 | 0.38 | [0.29, 0.48] | 7.9 | 0.45 |
|  | ESAT Negative *(m)* | <0.001 | -0.47 | [-0.58, -0.35] | -8 | -0.47 |

*Note:* Exploratory outcome variables at one-day follow-up compared to available baseline scores (ESAT and AWE-S) were analyzed via paired t-tests. ESAT factor outcomes at one-week and one-month post-intervention were similarly analyzed to determine if significant differences were sustained. Outcome reflects one-day follow-up unless indicated otherwise; *(w)* reflects one-week and *(m)* reflects one-month post-intervention outcomes. MD refers to mean difference error and *d* refers to effects size (Cohen’s d).
